# Determination of the spin and parity of all-charm tetraquarks

**DOI:** 10.1038/s41586-025-09711-7

**Published:** 2025-12-03

**Authors:** The CMS Collaboration, A. Hayrapetyan, V. Makarenko, A. Tumasyan, W. Adam, J. W. Andrejkovic, L. Benato, T. Bergauer, M. Dragicevic, C. Giordano, P. S. Hussain, M. Jeitler, N. Krammer, A. Li, D. Liko, M. Matthewman, I. Mikulec, J. Schieck, R. Schöfbeck, D. Schwarz, M. Shooshtari, M. Sonawane, W. Waltenberger, C.-E. Wulz, T. Janssen, H. Kwon, D. Ocampo Henao, T. Van Laer, P. Van Mechelen, J. Bierkens, N. Breugelmans, J. D’Hondt, S. Dansana, A. De Moor, M. Delcourt, F. Heyen, Y. Hong, P. Kashko, S. Lowette, I. Makarenko, D. Müller, J. Song, S. Tavernier, M. Tytgat, G. P. Van Onsem, S. Van Putte, D. Vannerom, B. Bilin, B. Clerbaux, A. K. Das, I. De Bruyn, G. De Lentdecker, H. Evard, L. Favart, P. Gianneios, A. Khalilzadeh, F. A. Khan, A. Malara, M. A. Shahzad, L. Thomas, M. Vanden Bemden, C. Vander Velde, P. Vanlaer, F. Zhang, M. De Coen, D. Dobur, G. Gokbulut, J. Knolle, L. Lambrecht, D. Marckx, K. Skovpen, N. Van Den Bossche, J. van der Linden, J. Vandenbroeck, L. Wezenbeek, S. Bein, A. Benecke, A. Bethani, G. Bruno, A. Cappati, J. De Favereau De Jeneret, C. Delaere, A. Giammanco, A. O. Guzel, V. Lemaitre, J. Lidrych, P. Malek, P. Mastrapasqua, S. Turkcapar, G. A. Alves, M. Barroso Ferreira Filho, E. Coelho, C. Hensel, T. Menezes De Oliveira, C. Mora Herrera, P. Rebello Teles, M. Soeiro, E. J. Tonelli Manganote, A. Vilela Pereira, W. L. Aldá Júnior, H. Brandao Malbouisson, W. Carvalho, J. Chinellato, M. Costa Reis, E. M. Da Costa, G. G. Da Silveira, D. De Jesus Damiao, S. Fonseca De Souza, R. Gomes De Souza, S. S. Jesus, T. Laux Kuhn, M. Macedo, K. Mota Amarilo, L. Mundim, H. Nogima, J. P. Pinheiro, A. Santoro, A. Sznajder, M. Thiel, F. Torres Da Silva De Araujo, C. A. Bernardes, T. R. Fernandez Perez Tomei, E. M. Gregores, B. Lopes Da Costa, I. Maietto Silverio, P. G. Mercadante, S. F. Novaes, B. Orzari, Sandra S. Padula, V. Scheurer, A. Aleksandrov, G. Antchev, P. Danev, R. Hadjiiska, P. Iaydjiev, M. Misheva, M. Shopova, G. Sultanov, A. Dimitrov, L. Litov, B. Pavlov, P. Petkov, A. Petrov, S. Keshri, D. Laroze, S. Thakur, W. Brooks, T. Cheng, T. Javaid, L. Wang, L. Yuan, Z. Hu, Z. Liang, J. Liu, X. Wang, G. M. Chen, H. S. Chen, M. Chen, Y. Chen, Q. Hou, X. Hou, F. Iemmi, C. H. Jiang, A. Kapoor, H. Liao, G. Liu, Z.-A. Liu, J. N. Song, S. Song, J. Tao, C. Wang, J. Wang, H. Zhang, J. Zhao, A. Agapitos, Y. Ban, A. Carvalho Antunes De Oliveira, S. Deng, B. Guo, Q. Guo, C. Jiang, A. Levin, C. Li, Q. Li, Y. Mao, S. Qian, S. J. Qian, X. Qin, X. Sun, D. Wang, J. Wang, H. Yang, M. Zhang, Y. Zhao, C. Zhou, S. Yang, Z. You, K. Jaffel, N. Lu, G. Bauer, Z. Cui, B. Li, H. Wang, K. Yi, J. Zhang, Y. Li, Z. Lin, C. Lu, M. Xiao, C. Avila, D. A. Barbosa Trujillo, A. Cabrera, C. Florez, J. Fraga, J. A. Reyes Vega, C. Rendón, M. Rodriguez, A. A. Ruales Barbosa, J. D. Ruiz Alvarez, N. Godinovic, D. Lelas, A. Sculac, M. Kovac, A. Petkovic, T. Sculac, P. Bargassa, V. Brigljevic, B. K. Chitroda, D. Ferencek, K. Jakovcic, A. Starodumov, T. Susa, A. Attikis, K. Christoforou, A. Hadjiagapiou, C. Leonidou, C. Nicolaou, L. Paizanos, F. Ptochos, P. A. Razis, H. Rykaczewski, H. Saka, A. Stepennov, M. Finger, M. Finger, E. Ayala, E. Carrera Jarrin, Y. Assran, B. El-mahdy, M. Abdullah Al-Mashad, A. Hussein, H. Mohammed, K. Ehataht, M. Kadastik, T. Lange, C. Nielsen, J. Pata, M. Raidal, N. Seeba, L. Tani, A. Milieva, K. Osterberg, M. Voutilainen, N. Bin Norjoharuddeen, E. Brücken, F. Garcia, P. Inkaew, K. T. S. Kallonen, R. Kumar Verma, T. Lampén, K. Lassila-Perini, B. Lehtela, S. Lehti, T. Lindén, N. R. Mancilla Xinto, M. Myllymäki, M. M. Rantanen, S. Saariokari, N. T. Toikka, J. Tuominiemi, H. Kirschenmann, P. Luukka, H. Petrow, M. Besancon, F. Couderc, M. Dejardin, D. Denegri, P. Devouge, J. L. Faure, F. Ferri, P. Gaigne, S. Ganjour, P. Gras, G. Hamel de Monchenault, M. Kumar, V. Lohezic, J. Malcles, F. Orlandi, L. Portales, S. Ronchi, M. Ö. Sahin, A. Savoy-Navarro, P. Simkina, M. Titov, M. Tornago, F. Beaudette, G. Boldrini, P. Busson, C. Charlot, M. Chiusi, T. D. Cuisset, F. Damas, O. Davignon, A. De Wit, T. Debnath, I. T. Ehle, B. A. Fontana Santos Alves, S. Ghosh, A. Gilbert, R. Granier de Cassagnac, L. Kalipoliti, M. Manoni, M. Nguyen, S. Obraztsov, C. Ochando, R. Salerno, J. B. Sauvan, Y. Sirois, G. Sokmen, L. Urda Gómez, A. Zabi, A. Zghiche, J.-L. Agram, J. Andrea, D. Bloch, J.-M. Brom, E. C. Chabert, C. Collard, G. Coulon, S. Falke, U. Goerlach, R. Haeberle, A.-C. Le Bihan, M. Meena, O. Poncet, G. Saha, P. Vaucelle, A. Di Florio, D. Amram, S. Beauceron, B. Blancon, G. Boudoul, N. Chanon, D. Contardo, P. Depasse, C. Dozen, H. El Mamouni, J. Fay, S. Gascon, M. Gouzevitch, C. Greenberg, G. Grenier, B. Ille, E. Jourd’huy, I. B. Laktineh, M. Lethuillier, B. Massoteau, L. Mirabito, A. Purohit, M. Vander Donckt, J. Xiao, I. Lomidze, T. Toriashvili, Z. Tsamalaidze, V. Botta, S. Consuegra Rodríguez, L. Feld, K. Klein, M. Lipinski, D. Meuser, P. Nattland, V. Oppenländer, A. Pauls, D. Pérez Adán, N. Röwert, M. Teroerde, C. Daumann, S. Diekmann, A. Dodonova, N. Eich, D. Eliseev, F. Engelke, J. Erdmann, M. Erdmann, B. Fischer, T. Hebbeker, K. Hoepfner, F. Ivone, A. Jung, N. Kumar, M. Y. Lee, F. Mausolf, M. Merschmeyer, A. Meyer, F. Nowotny, A. Pozdnyakov, W. Redjeb, H. Reithler, U. Sarkar, V. Sarkisovi, A. Schmidt, C. Seth, A. Sharma, J. L. Spah, V. Vaulin, S. Zaleski, M. R. Beckers, C. Dziwok, G. Flügge, N. Hoeflich, T. Kress, A. Nowack, O. Pooth, A. Stahl, A. Zotz, H. Aarup Petersen, A. Abel, M. Aldaya Martin, J. Alimena, S. Amoroso, Y. An, I. Andreev, J. Bach, S. Baxter, M. Bayatmakou, H. Becerril Gonzalez, O. Behnke, A. Belvedere, F. Blekman, K. Borras, A. Campbell, S. Chatterjee, L. X. Coll Saravia, G. Eckerlin, D. Eckstein, E. Gallo, A. Geiser, V. Guglielmi, M. Guthoff, A. Hinzmann, L. Jeppe, M. Kasemann, C. Kleinwort, R. Kogler, M. Komm, D. Krücker, W. Lange, D. Leyva Pernia, K.-Y. Lin, K. Lipka, W. Lohmann, J. Malvaso, R. Mankel, I.-A. Melzer-Pellmann, M. Mendizabal Morentin, A. B. Meyer, G. Milella, K. Moral Figueroa, A. Mussgiller, L. P. Nair, J. Niedziela, A. Nürnberg, J. Park, E. Ranken, A. Raspereza, D. Rastorguev, L. Rygaard, M. Scham, S. Schnake, P. Schütze, C. Schwanenberger, D. Selivanova, K. Sharko, M. Shchedrolosiev, D. Stafford, M. Torkian, F. Vazzoler, A. Ventura Barroso, R. Walsh, D. Wang, Q. Wang, K. Wichmann, L. Wiens, C. Wissing, Y. Yang, S. Zakharov, A. Zimermmane Castro Santos, A. Albrecht, A. R. Alves Andrade, M. Antonello, S. Bollweg, M. Bonanomi, K. El Morabit, Y. Fischer, M. Frahm, E. Garutti, A. Grohsjean, A. A. Guvenli, J. Haller, D. Hundhausen, G. Kasieczka, P. Keicher, R. Klanner, W. Korcari, T. Kramer, C. C. Kuo, F. Labe, J. Lange, A. Lobanov, L. Moureaux, M. Mrowietz, A. Nigamova, K. Nikolopoulos, Y. Nissan, A. Paasch, K. J. Pena Rodriguez, N. Prouvost, T. Quadfasel, B. Raciti, M. Rieger, D. Savoiu, P. Schleper, M. Schröder, J. Schwandt, M. Sommerhalder, H. Stadie, G. Steinbrück, A. Tews, R. Ward, B. Wiederspan, M. Wolf, S. Brommer, E. Butz, Y. M. Chen, T. Chwalek, A. Dierlamm, G. G. Dincer, U. Elicabuk, N. Faltermann, M. Giffels, A. Gottmann, F. Hartmann, R. Hofsaess, M. Horzela, U. Husemann, J. Kieseler, M. Klute, R. Kunnilan Muhammed Rafeek, O. Lavoryk, J. M. Lawhorn, A. Lintuluoto, S. Maier, M. Mormile, Th. Müller, E. Pfeffer, M. Presilla, G. Quast, K. Rabbertz, B. Regnery, R. Schmieder, N. Shadskiy, I. Shvetsov, H. J. Simonis, L. Sowa, L. Stockmeier, K. Tauqeer, M. Toms, B. Topko, N. Trevisani, C. Verstege, T. Voigtländer, R. F. Von Cube, J. Von Den Driesch, M. Wassmer, R. Wolf, W. D. Zeuner, X. Zuo, G. Anagnostou, G. Daskalakis, A. Kyriakis, G. Melachroinos, Z. Painesis, I. Paraskevas, N. Saoulidou, K. Theofilatos, E. Tziaferi, K. Vellidis, I. Zisopoulos, T. Chatzistavrou, G. Karapostoli, K. Kousouris, E. Siamarkou, G. Tsipolitis, I. Bestintzanos, I. Evangelou, C. Foudas, P. Katsoulis, P. Kokkas, P. G. Kosmoglou Kioseoglou, N. Manthos, I. Papadopoulos, J. Strologas, D. Druzhkin, C. Hajdu, D. Horvath, K. Márton, A. J. Rádl, F. Sikler, V. Veszpremi, M. Csanád, K. Farkas, A. Fehérkuti, M. M. A. Gadallah, Á. Kadlecsik, M. León Coello, G. Pásztor, G. I. Veres, B. Ujvari, G. Zilizi, G. Bencze, S. Czellar, J. Molnar, Z. Szillasi, T. Csorgo, F. Nemes, T. Novak, I. Szanyi, S. Bansal, S. B. Beri, V. Bhatnagar, G. Chaudhary, S. Chauhan, N. Dhingra, A. Kaur, H. Kaur, M. Kaur, S. Kumar, T. Sheokand, J. B. Singh, A. Singla, A. Bhardwaj, A. Chhetri, B. C. Choudhary, A. Kumar, M. Naimuddin, S. Phor, K. Ranjan, M. K. Saini, S. Acharya, B. Gomber, B. Sahu, S. Mukherjee, S. Baradia, S. Bhattacharya, S. Das Gupta, S. Dutta, S. Sarkar, M. M. Ameen, P. K. Behera, S. Chatterjee, G. Dash, A. Dattamunsi, P. Jana, P. Kalbhor, S. Kamble, J. R. Komaragiri, T. Mishra, P. R. Pujahari, A. K. Sikdar, R. K. Singh, P. Verma, S. Verma, A. Vijay, B. K. Sirasva, L. Bhatt, S. Dugad, G. B. Mohanty, M. Shelake, P. Suryadevara, A. Bala, S. Banerjee, S. Barman, R. M. Chatterjee, M. Guchait, Sh. Jain, A. Jaiswal, B. M. Joshi, S. Kumar, M. Maity, G. Majumder, K. Mazumdar, S. Parolia, R. Saxena, A. Thachayath, S. Bahinipati, D. Maity, P. Mal, K. Naskar, A. Nayak, S. Nayak, K. Pal, R. Raturi, P. Sadangi, S. K. Swain, S. Varghese, D. Vats, A. Alpana, S. Dube, P. Hazarika, B. Kansal, A. Laha, R. Sharma, S. Sharma, K. Y. Vaish, S. Ghosh, H. Bakhshiansohi, A. Jafari, V. Sedighzadeh Dalavi, M. Zeinali, S. Bashiri, S. Chenarani, S. M. Etesami, Y. Hosseini, M. Khakzad, E. Khazaie, M. Mohammadi Najafabadi, S. Tizchang, M. Felcini, M. Grunewald, M. Abbrescia, M. Barbieri, M. Buonsante, A. Colaleo, D. Creanza, B. D’Anzi, N. De Filippis, M. De Palma, W. Elmetenawee, N. Ferrara, L. Fiore, L. Longo, M. Louka, G. Maggi, M. Maggi, I. Margjeka, V. Mastrapasqua, S. My, F. Nenna, S. Nuzzo, A. Pellecchia, A. Pompili, G. Pugliese, R. Radogna, D. Ramos, A. Ranieri, L. Silvestris, F. M. Simone, Ü. Sözbilir, A. Stamerra, D. Troiano, R. Venditti, P. Verwilligen, A. Zaza, G. Abbiendi, C. Battilana, D. Bonacorsi, P. Capiluppi, F. R. Cavallo, G. M. Dallavalle, T. Diotalevi, F. Fabbri, A. Fanfani, D. Fasanella, P. Giacomelli, C. Grandi, L. Guiducci, S. Lo Meo, M. Lorusso, L. Lunerti, S. Marcellini, G. Masetti, F. L. Navarria, G. Paggi, A. Perrotta, F. Primavera, A. M. Rossi, S. Rossi Tisbeni, T. Rovelli, G. P. Siroli, S. Costa, A. Di Mattia, A. Lapertosa, R. Potenza, A. Tricomi, J. Altork, P. Assiouras, G. Barbagli, G. Bardelli, M. Bartolini, A. Calandri, B. Camaiani, A. Cassese, R. Ceccarelli, V. Ciulli, C. Civinini, R. D’Alessandro, L. Damenti, E. Focardi, T. Kello, G. Latino, P. Lenzi, M. Lizzo, M. Meschini, S. Paoletti, A. Papanastassiou, G. Sguazzoni, L. Viliani, L. Benussi, S. Colafranceschi, S. Meola, D. Piccolo, M. Alves Gallo Pereira, F. Ferro, E. Robutti, S. Tosi, A. Benaglia, F. Brivio, V. Camagni, F. Cetorelli, F. De Guio, M. E. Dinardo, P. Dini, S. Gennai, R. Gerosa, A. Ghezzi, P. Govoni, L. Guzzi, M. R. Kim, G. Lavizzari, M. T. Lucchini, M. Malberti, S. Malvezzi, A. Massironi, D. Menasce, L. Moroni, M. Paganoni, S. Palluotto, D. Pedrini, A. Perego, B. S. Pinolini, G. Pizzati, S. Ragazzi, T. Tabarelli de Fatis, S. Buontempo, C. Di Fraia, F. Fabozzi, L. Favilla, A. O. M. Iorio, L. Lista, P. Paolucci, B. Rossi, P. Azzi, N. Bacchetta, D. Bisello, P. Bortignon, G. Bortolato, A. C. M. Bulla, P. Checchia, T. Dorigo, F. Gasparini, U. Gasparini, S. Giorgetti, E. Lusiani, M. Margoni, A. T. Meneguzzo, F. Montecassiano, J. Pazzini, P. Ronchese, R. Rossin, F. Simonetto, M. Tosi, A. Triossi, S. Ventura, M. Zanetti, P. Zotto, A. Zucchetta, A. Braghieri, S. Calzaferri, P. Montagna, M. Pelliccioni, V. Re, C. Riccardi, P. Salvini, I. Vai, P. Vitulo, S. Ajmal, M. E. Ascioti, G. M. Bilei, C. Carrivale, D. Ciangottini, L. Della Penna, L. Fanò, V. Mariani, M. Menichelli, F. Moscatelli, A. Rossi, A. Santocchia, D. Spiga, T. Tedeschi, C. Aimè, C. A. Alexe, P. Asenov, P. Azzurri, G. Bagliesi, R. Bhattacharya, L. Bianchini, T. Boccali, E. Bossini, D. Bruschini, L. Calligaris, R. Castaldi, F. Cattafesta, M. A. Ciocci, M. Cipriani, R. Dell’Orso, S. Donato, R. Forti, A. Giassi, F. Ligabue, A. C. Marini, D. Matos Figueiredo, A. Messineo, S. Mishra, V. K. Muraleedharan Nair Bindhu, S. Nandan, F. Palla, M. Riggirello, A. Rizzi, G. Rolandi, S. Roy Chowdhury, T. Sarkar, A. Scribano, P. Solanki, P. Spagnolo, F. Tenchini, R. Tenchini, G. Tonelli, N. Turini, F. Vaselli, A. Venturi, P. G. Verdini, P. Akrap, C. Basile, S. C. Behera, F. Cavallari, L. Cunqueiro Mendez, F. De Riggi, D. Del Re, E. Di Marco, M. Diemoz, F. Errico, L. Frosina, R. Gargiulo, B. Harikrishnan, F. Lombardi, E. Longo, L. Martikainen, J. Mijuskovic, G. Organtini, N. Palmeri, R. Paramatti, C. Quaranta, S. Rahatlou, C. Rovelli, F. Santanastasio, L. Soffi, V. Vladimirov, N. Amapane, R. Arcidiacono, S. Argiro, M. Arneodo, N. Bartosik, R. Bellan, A. Bellora, C. Biino, C. Borca, N. Cartiglia, M. Costa, R. Covarelli, N. Demaria, L. Finco, M. Grippo, B. Kiani, L. Lanteri, F. Legger, F. Luongo, C. Mariotti, S. Maselli, A. Mecca, L. Menzio, P. Meridiani, E. Migliore, M. Monteno, M. M. Obertino, G. Ortona, L. Pacher, N. Pastrone, M. Ruspa, F. Siviero, V. Sola, A. Solano, A. Staiano, C. Tarricone, D. Trocino, G. Umoret, E. Vlasov, R. White, J. Babbar, S. Belforte, V. Candelise, M. Casarsa, F. Cossutti, K. De Leo, G. Della Ricca, R. Delli Gatti, S. Dogra, J. Hong, J. Kim, T. Kim, D. Lee, H. Lee, J. Lee, S. W. Lee, C. S. Moon, Y. D. Oh, S. Sekmen, B. Tae, Y. C. Yang, M. S. Kim, G. Bak, P. Gwak, H. Kim, D. H. Moon, J. Seo, E. Asilar, F. Carnevali, J. Choi, T. J. Kim, Y. Ryou, S. Ha, S. Han, B. Hong, K. Lee, K. S. Lee, S. Lee, J. Yoo, J. Goh, J. Shin, S. Yang, Y. Kang, H. S. Kim, Y. Kim, S. Lee, J. Almond, J. H. Bhyun, J. Choi, W. Jun, H. Kim, J. Kim, T. Kim, Y. Kim, Y. W. Kim, S. Ko, H. Lee, J. Lee, B. H. Oh, S. B. Oh, J. Shin, U. K. Yang, I. Yoon, W. Jang, D. Y. Kang, D. Kim, S. Kim, B. Ko, J. S. H. Lee, Y. Lee, I. C. Park, Y. Roh, I. J. Watson, G. Cho, K. Hwang, B. Kim, S. Kim, K. Lee, H. D. Yoo, M. Choi, Y. Lee, I. Yu, T. Beyrouthy, Y. Gharbia, F. Alazemi, K. Dreimanis, O. M. Eberlins, A. Gaile, C. Munoz Diaz, D. Osite, G. Pikurs, R. Plese, A. Potrebko, M. Seidel, D. Sidiropoulos Kontos, N. R. Strautnieks, M. Ambrozas, A. Juodagalvis, S. Nargelas, A. Rinkevicius, G. Tamulaitis, I. Yusuff, Z. Zolkapli, J. F. Benitez, A. Castaneda Hernandez, A. Cota Rodriguez, L. E. Cuevas Picos, H. A. Encinas Acosta, L. G. Gallegos Maríñez, J. A. Murillo Quijada, A. Sehrawat, L. Valencia Palomo, G. Ayala, H. Castilla-Valdez, H. Crotte Ledesma, R. Lopez-Fernandez, J. Mejia Guisao, R. Reyes-Almanza, A. Sánchez Hernández, C. Oropeza Barrera, D. L. Ramirez Guadarrama, M. Ramírez García, I. Bautista, F. E. Neri Huerta, I. Pedraza, H. A. Salazar Ibarguen, C. Uribe Estrada, I. Bubanja, N. Raicevic, P. H. Butler, A. Ahmad, M. I. Asghar, A. Awais, M. I. M. Awan, W. A. Khan, V. Avati, L. Forthomme, L. Grzanka, M. Malawski, K. Piotrzkowski, M. Bluj, M. Górski, M. Kazana, M. Szleper, P. Zalewski, K. Bunkowski, K. Doroba, A. Kalinowski, M. Konecki, J. Krolikowski, A. Muhammad, P. Fokow, K. Pozniak, W. Zabolotny, M. Araujo, D. Bastos, C. Beirão Da Cruz E Silva, A. Boletti, M. Bozzo, T. Camporesi, G. Da Molin, M. Gallinaro, J. Hollar, N. Leonardo, G. B. Marozzo, A. Petrilli, M. Pisano, J. Seixas, J. Varela, J. W. Wulff, P. Adzic, L. Markovic, P. Milenovic, V. Milosevic, D. Devetak, M. Dordevic, J. Milosevic, L. Nadderd, V. Rekovic, M. Stojanovic, M. Alcalde Martinez, J. Alcaraz Maestre, Cristina F. Bedoya, J. A. Brochero Cifuentes, Oliver M. Carretero, M. Cepeda, M. Cerrada, N. Colino, J. Cuchillo Ortega, B. De La Cruz, A. Delgado Peris, A. Escalante Del Valle, D. Fernández Del Val, J. P. Fernández Ramos, J. Flix, M. C. Fouz, M. Gonzalez Hernandez, O. Gonzalez Lopez, S. Goy Lopez, J. M. Hernandez, M. I. Josa, J. Llorente Merino, C. Martin Perez, E. Martin Viscasillas, D. Moran, C. M. Morcillo Perez, R. Paz Herrera, C. Perez Dengra, A. Pérez-Calero Yzquierdo, J. Puerta Pelayo, I. Redondo, J. Vazquez Escobar, J. F. de Trocóniz, B. Alvarez Gonzalez, J. Ayllon Torresano, A. Cardini, J. Cuevas, J. Del Riego Badas, D. Estrada Acevedo, J. Fernandez Menendez, S. Folgueras, I. Gonzalez Caballero, P. Leguina, M. Obeso Menendez, E. Palencia Cortezon, J. Prado Pico, A. Soto Rodríguez, C. Vico Villalba, P. Vischia, S. Blanco Fernández, I. J. Cabrillo, A. Calderon, J. Duarte Campderros, M. Fernandez, G. Gomez, C. Lasaosa García, R. Lopez Ruiz, C. Martinez Rivero, P. Martinez Ruiz del Arbol, F. Matorras, P. Matorras Cuevas, E. Navarrete Ramos, J. Piedra Gomez, C. Quintana San Emeterio, L. Scodellaro, I. Vila, R. Vilar Cortabitarte, J. M. Vizan Garcia, B. Kailasapathy, D. D. C. Wickramarathna, W. G. D. Dharmaratna, K. Liyanage, N. Perera, D. Abbaneo, C. Amendola, R. Ardino, E. Auffray, J. Baechler, D. Barney, M. Bianco, A. Bocci, L. Borgonovi, C. Botta, A. Bragagnolo, C. E. Brown, C. Caillol, G. Cerminara, P. Connor, D. d’Enterria, A. Dabrowski, A. David, A. De Roeck, M. M. Defranchis, M. Deile, M. Dobson, W. Funk, A. Gaddi, S. Giani, D. Gigi, K. Gill, F. Glege, M. Glowacki, A. Gruber, J. Hegeman, J. K. Heikkilä, B. Huber, V. Innocente, T. James, P. Janot, O. Kaluzinska, O. Karacheban, G. Karathanasis, S. Laurila, P. Lecoq, C. Lourenço, A.-M. Lyon, M. Magherini, L. Malgeri, M. Mannelli, A. Mehta, F. Meijers, J. A. Merlin, S. Mersi, E. Meschi, M. Migliorini, F. Monti, F. Moortgat, M. Mulders, M. Musich, I. Neutelings, S. Orfanelli, F. Pantaleo, M. Pari, G. Petrucciani, A. Pfeiffer, M. Pierini, M. Pitt, H. Qu, D. Rabady, B. Ribeiro Lopes, F. Riti, P. Rosado, M. Rovere, H. Sakulin, R. Salvatico, S. Sanchez Cruz, S. Scarfi, C. Schwick, M. Selvaggi, A. Sharma, K. Shchelina, P. Silva, P. Sphicas, A. G. Stahl Leiton, A. Steen, S. Summers, D. Treille, P. Tropea, E. Vernazza, J. Wanczyk, J. Wang, S. Wuchterl, M. Zarucki, P. Zehetner, P. Zejdl, G. Zevi Della Porta, T. Bevilacqua, L. Caminada, W. Erdmann, R. Horisberger, Q. Ingram, H. C. Kaestli, D. Kotlinski, C. Lange, U. Langenegger, M. Missiroli, L. Noehte, T. Rohe, A. Samalan, T. K. Aarrestad, M. Backhaus, G. Bonomelli, C. Cazzaniga, K. Datta, P. De Bryas Dexmiers D’archiacchiac, A. De Cosa, G. Dissertori, M. Dittmar, M. Donegà, F. Eble, K. Gedia, F. Glessgen, C. Grab, N. Härringer, T. G. Harte, W. Lustermann, M. Malucchi, R. A. Manzoni, M. Marchegiani, L. Marchese, A. Mascellani, F. Nessi-Tedaldi, F. Pauss, V. Perovic, B. Ristic, R. Seidita, J. Steggemann, A. Tarabini, D. Valsecchi, R. Wallny, C. Amsler, P. Bärtschi, F. Bilandzija, M. F. Canelli, G. Celotto, K. Cormier, M. Huwiler, W. Jin, A. Jofrehei, B. Kilminster, T. H. Kwok, S. Leontsinis, V. Lukashenko, A. Macchiolo, F. Meng, J. Motta, A. Reimers, P. Robmann, M. Senger, E. Shokr, F. Stäger, R. Tramontano, D. Bhowmik, C. M. Kuo, P. K. Rout, S. Taj, P. C. Tiwari, L. Ceard, K. F. Chen, Z. G. Chen, A. De Iorio, W.-S. Hou, T. H. Hsu, Y. W. Kao, S. Karmakar, G. Kole, Y. Y. Li, R.-S. Lu, E. Paganis, X. F. Su, J. Thomas-Wilsker, L. S. Tsai, D. Tsionou, H. Y. Wu, E. Yazgan, C. Asawatangtrakuldee, N. Srimanobhas, Y. Maghrbi, D. Agyel, F. Dolek, I. Dumanoglu, Y. Guler, E. Gurpinar Guler, C. Isik, O. Kara, A. Kayis Topaksu, Y. Komurcu, G. Onengut, K. Ozdemir, B. Tali, U. G. Tok, E. Uslan, I. S. Zorbakir, M. Yalvac, B. Akgun, I. O. Atakisi, E. Gülmez, M. Kaya, O. Kaya, M. A. Sarkisla, S. Tekten, A. Cakir, K. Cankocak, S. Sen, O. Aydilek, B. Hacisahinoglu, I. Hos, B. Kaynak, S. Ozkorucuklu, O. Potok, H. Sert, C. Simsek, C. Zorbilmez, S. Cerci, B. Isildak, E. Simsek, D. Sunar Cerci, T. Yetkin, A. Boyaryntsev, O. Dadazhanova, B. Grynyov, L. Levchuk, J. J. Brooke, A. Bundock, F. Bury, E. Clement, D. Cussans, D. Dharmender, H. Flacher, J. Goldstein, H. F. Heath, M.-L. Holmberg, L. Kreczko, S. Paramesvaran, L. Robertshaw, M. S. Sanjrani, J. Segal, V. J. Smith, A. H. Ball, K. W. Bell, A. Belyaev, C. Brew, R. M. Brown, D. J. A. Cockerill, A. Elliot, K. V. Ellis, J. Gajownik, K. Harder, S. Harper, J. Linacre, K. Manolopoulos, M. Moallemi, D. M. Newbold, E. Olaiya, D. Petyt, T. Reis, A. R. Sahasransu, G. Salvi, T. Schuh, C. H. Shepherd-Themistocleous, I. R. Tomalin, K. C. Whalen, T. Williams, I. Andreou, R. Bainbridge, P. Bloch, O. Buchmuller, C. A. Carrillo Montoya, D. Colling, J. S. Dancu, I. Das, P. Dauncey, G. Davies, M. Della Negra, S. Fayer, G. Fedi, G. Hall, H. R. Hoorani, A. Howard, G. Iles, C. R. Knight, P. Krueper, J. Langford, K. H. Law, J. León Holgado, E. Leutgeb, L. Lyons, A.-M. Magnan, B. Maier, S. Mallios, A. Mastronikolis, M. Mieskolainen, J. Nash, M. Pesaresi, P. B. Pradeep, B. C. Radburn-Smith, A. Richards, A. Rose, L. Russell, K. Savva, C. Seez, R. Shukla, A. Tapper, K. Uchida, G. P. Uttley, T. Virdee, M. Vojinovic, N. Wardle, D. Winterbottom, J. E. Cole, A. Khan, P. Kyberd, I. D. Reid, S. Abdullin, A. Brinkerhoff, E. Collins, M. R. Darwish, J. Dittmann, K. Hatakeyama, V. Hegde, J. Hiltbrand, B. McMaster, J. Samudio, S. Sawant, C. Sutantawibul, J. Wilson, J. M. Hogan, R. Bartek, A. Dominguez, S. Raj, A. E. Simsek, S. S. Yu, B. Bam, A. Buchot Perraguin, S. Campbell, R. Chudasama, S. I. Cooper, C. Crovella, G. Fidalgo, S. V. Gleyzer, A. Khukhunaishvili, K. Matchev, E. Pearson, C. U. Perez, P. Rumerio, E. Usai, R. Yi, S. Cholak, G. De Castro, Z. Demiragli, C. Erice, C. Fangmeier, C. Fernandez Madrazo, E. Fontanesi, J. Fulcher, F. Golf, S. Jeon, J. O’Cain, I. Reed, J. Rohlf, K. Salyer, D. Sperka, D. Spitzbart, I. Suarez, A. Tsatsos, E. Wurtz, A. G. Zecchinelli, G. Barone, G. Benelli, D. Cutts, S. Ellis, L. Gouskos, M. Hadley, U. Heintz, K. W. Ho, T. Kwon, G. Landsberg, K. T. Lau, J. Luo, S. Mondal, J. Roloff, T. Russell, S. Sagir, X. Shen, M. Stamenkovic, N. Venkatasubramanian, S. Abbott, B. Barton, R. Breedon, H. Cai, M. Calderon De La Barca Sanchez, M. Chertok, M. Citron, J. Conway, P. T. Cox, R. Erbacher, O. Kukral, G. Mocellin, S. Ostrom, I. Salazar Segovia, W. Wei, S. Yoo, K. Adamidis, M. Bachtis, D. Campos, R. Cousins, A. Datta, G. Flores Avila, J. Hauser, M. Ignatenko, M. A. Iqbal, T. Lam, Y. F. Lo, E. Manca, A. Nunez Del Prado, D. Saltzberg, V. Valuev, R. Clare, J. W. Gary, G. Hanson, A. Aportela, A. Arora, J. G. Branson, S. Cittolin, S. Cooperstein, D. Diaz, J. Duarte, L. Giannini, Y. Gu, J. Guiang, V. Krutelyov, R. Lee, J. Letts, H. Li, M. Masciovecchio, F. Mokhtar, S. Mukherjee, M. Pieri, D. Primosch, M. Quinnan, V. Sharma, M. Tadel, E. Vourliotis, F. Würthwein, A. Yagil, Z. Zhao, A. Barzdukas, L. Brennan, C. Campagnari, S. Carron Montero, K. Downham, C. Grieco, M. M. Hussain, J. Incandela, J. Kim, M. W. K. Lai, A. J. Li, P. Masterson, J. Richman, S. N. Santpur, U. Sarica, R. Schmitz, F. Setti, J. Sheplock, D. Stuart, T. Á. Vámi, X. Yan, D. Zhang, A. Albert, S. Bhattacharya, A. Bornheim, O. Cerri, R. Kansal, J. Mao, H. B. Newman, G. Reales Gutiérrez, T. Sievert, M. Spiropulu, J. R. Vlimant, R. A. Wynne, S. Xie, J. Alison, S. An, M. Cremonesi, V. Dutta, E. Y. Ertorer, T. Ferguson, T. A. Giómez Espinosa, A. Harilal, A. Kallil Tharayil, M. Kanemura, C. Liu, P. Meiring, T. Mudholkar, S. Murthy, P. Palit, K. Park, M. Paulini, A. Roberts, A. Sanchez, W. Terrill, J. P. Cumalat, W. T. Ford, A. Hart, A. Hassani, S. Kwan, J. Pearkes, C. Savard, N. Schonbeck, K. Stenson, K. A. Ulmer, S. R. Wagner, N. Zipper, D. Zuolo, J. Alexander, X. Chen, D. J. Cranshaw, J. Dickinson, J. Fan, X. Fan, J. Grassi, S. Hogan, P. Kotamnives, J. Monroy, G. Niendorf, M. Oshiro, J. R. Patterson, M. Reid, A. Ryd, J. Thom, P. Wittich, R. Zou, L. Zygala, M. Albrow, M. Alyari, O. Amram, G. Apollinari, A. Apresyan, L. A. T. Bauerdick, D. Berry, J. Berryhill, P. C. Bhat, K. Burkett, J. N. Butler, A. Canepa, G. B. Cerati, H. W. K. Cheung, F. Chlebana, C. Cosby, G. Cummings, I. Dutta, V. D. Elvira, J. Freeman, A. Gandrakota, Z. Gecse, L. Gray, D. Green, A. Grummer, S. Grünendahl, D. Guerrero, O. Gutsche, R. M. Harris, T. C. Herwig, J. Hirschauer, B. Jayatilaka, S. Jindariani, M. Johnson, U. Joshi, T. Klijnsma, B. Klima, K. H. M. Kwok, S. Lammel, C. Lee, D. Lincoln, R. Lipton, T. Liu, K. Maeshima, D. Mason, P. McBride, P. Merkel, S. Mrenna, S. Nahn, J. Ngadiuba, D. Noonan, S. Norberg, V. Papadimitriou, N. Pastika, K. Pedro, C. Pena, C. E. Perez Lara, F. Ravera, A. Reinsvold Hall, L. Ristori, M. Safdari, E. Sexton-Kennedy, N. Smith, A. Soha, L. Spiegel, S. Stoynev, J. Strait, L. Taylor, S. Tkaczyk, N. V. Tran, L. Uplegger, E. W. Vaandering, C. Wang, I. Zoi, C. Aruta, P. Avery, D. Bourilkov, P. Chang, V. Cherepanov, R. D. Field, C. Huh, E. Koenig, M. Kolosova, J. Konigsberg, A. Korytov, N. Menendez, G. Mitselmakher, K. Mohrman, A. Muthirakalayil Madhu, N. Rawal, S. Rosenzweig, V. Sulimov, Y. Takahashi, J. Wang, T. Adams, A. Al Kadhim, A. Askew, S. Bower, R. Hashmi, R. S. Kim, T. Kolberg, G. Martinez, M. Mazza, H. Prosper, P. R. Prova, M. Wulansatiti, R. Yohay, B. Alsufyani, S. Butalla, S. Das, M. Hohlmann, M. Lavinsky, E. Yanes, M. R. Adams, N. Barnett, A. Baty, C. Bennett, R. Cavanaugh, R. Escobar Franco, O. Evdokimov, C. E. Gerber, H. Gupta, M. Hawksworth, A. Hingrajiya, D. J. Hofman, J. H. Lee, D. S. Lemos, C. Mills, S. Nanda, G. Nigmatkulov, B. Ozek, T. Phan, D. Pilipovic, R. Pradhan, E. Prifti, P. Roy, T. Roy, N. Singh, M. B. Tonjes, N. Varelas, M. A. Wadud, J. Yoo, M. Alhusseini, D. Blend, K. Dilsiz, O. K. Köseyan, A. Mestvirishvili, O. Neogi, H. Ogul, Y. Onel, A. Penzo, C. Snyder, E. Tiras, B. Blumenfeld, J. Davis, A. V. Gritsan, Z. Huang, L. Kang, S. Kyriacou, P. Maksimovic, M. Roguljic, S. Sekhar, M. V. Srivastav, M. Swartz, C. You, A. Abreu, L. F. Alcerro Alcerro, J. Anguiano, S. Arteaga Escatel, P. Baringer, A. Bean, Z. Flowers, D. Grove, J. King, G. Krintiras, M. Lazarovits, C. LE Mahieu, J. Marquez, M. Murray, M. Nickel, S. Popescu, C. Rogan, C. Royon, S. Rudrabhatla, S. Sanders, C. Smith, G. Wilson, B. Allmond, R. Gujju Gurunadha, N. Islam, A. Ivanov, K. Kaadze, Y. Maravin, J. Natoli, D. Roy, G. Sorrentino, A. Baden, A. Belloni, J. Bistany-riebman, S. C. Eno, N. J. Hadley, S. Jabeen, R. G. Kellogg, T. Koeth, B. Kronheim, S. Lascio, P. Major, A. C. Mignerey, C. Palmer, C. Papageorgakis, M. M. Paranjpe, E. Popova, A. Shevelev, L. Zhang, C. Baldenegro Barrera, J. Bendavid, H. Bossi, S. Bright-Thonney, I. A. Cali, Y. C. Chen, P. C. Chou, M. D’Alfonso, J. Eysermans, C. Freer, G. Gomez-Ceballos, M. Goncharov, G. Grosso, P. Harris, D. Hoang, G. M. Innocenti, D. Kovalskyi, J. Krupa, L. Lavezzo, Y.-J. Lee, K. Long, C. Mcginn, A. Novak, M. I. Park, C. Paus, C. Reissel, C. Roland, G. Roland, S. Rothman, T. A. Sheng, G. S. F. Stephans, D. Walter, Z. Wang, B. Wyslouch, T. J. Yang, B. Crossman, W. J. Jackson, C. Kapsiak, M. Krohn, D. Mahon, J. Mans, B. Marzocchi, R. Rusack, O. Sancar, R. Saradhy, N. Strobbe, K. Bloom, D. R. Claes, G. Haza, J. Hossain, C. Joo, I. Kravchenko, A. Rohilla, J. E. Siado, W. Tabb, A. Vagnerini, A. Wightman, F. Yan, H. Bandyopadhyay, L. Hay, H. W. Hsia, I. Iashvili, A. Kalogeropoulos, A. Kharchilava, A. Mandal, M. Morris, D. Nguyen, S. Rappoccio, H. Rejeb Sfar, A. Williams, P. Young, D. Yu, G. Alverson, E. Barberis, J. Bonilla, B. Bylsma, M. Campana, J. Dervan, Y. Haddad, Y. Han, I. Israr, A. Krishna, M. Lu, N. Manganelli, R. Mccarthy, D. M. Morse, T. Orimoto, A. Parker, L. Skinnari, C. S. Thoreson, E. Tsai, D. Wood, S. Dittmer, K. A. Hahn, Y. Liu, M. Mcginnis, Y. Miao, D. G. Monk, M. H. Schmitt, A. Taliercio, M. Velasco, J. Wang, G. Agarwal, R. Band, R. Bucci, S. Castells, A. Das, A. Ehnis, R. Goldouzian, M. Hildreth, K. Hurtado Anampa, T. Ivanov, C. Jessop, A. Karneyeu, K. Lannon, J. Lawrence, N. Loukas, L. Lutton, J. Mariano, N. Marinelli, I. Mcalister, T. McCauley, C. Mcgrady, C. Moore, Y. Musienko, H. Nelson, M. Osherson, A. Piccinelli, R. Ruchti, A. Townsend, Y. Wan, M. Wayne, H. Yockey, A. Basnet, M. Carrigan, R. De Los Santos, L. S. Durkin, C. Hill, M. Joyce, M. Nunez Ornelas, D. A. Wenzl, B. L. Winer, B. R. Yates, H. Bouchamaoui, K. Coldham, P. Das, G. Dezoort, P. Elmer, A. Frankenthal, M. Galli, B. Greenberg, N. Haubrich, K. Kennedy, G. Kopp, Y. Lai, D. Lange, A. Loeliger, D. Marlow, I. Ojalvo, J. Olsen, F. Simpson, D. Stickland, C. Tully, S. Malik, R. Sharma, S. Chandra, R. Chawla, A. Gu, L. Gutay, M. Jones, A. W. Jung, D. Kondratyev, M. Liu, G. Negro, N. Neumeister, G. Paspalaki, S. Piperov, N. R. Saha, J. F. Schulte, F. Wang, A. Wildridge, W. Xie, Y. Yao, Y. Zhong, N. Parashar, A. Pathak, E. Shumka, D. Acosta, A. Agrawal, C. Arbour, T. Carnahan, K. M. Ecklund, P. J. Fernández Manteca, S. Freed, P. Gardner, F. J. M. Geurts, T. Huang, I. Krommydas, N. Lewis, W. Li, J. Lin, O. Miguel Colin, B. P. Padley, R. Redjimi, J. Rotter, E. Yigitbasi, Y. Zhang, O. Bessidskaia Bylund, A. Bodek, P. de Barbaro, R. Demina, A. Garcia-Bellido, H. S. Hare, O. Hindrichs, N. Parmar, P. Parygin, H. Seo, R. Taus, B. Chiarito, J. P. Chou, S. V. Clark, S. Donnelly, D. Gadkari, Y. Gershtein, E. Halkiadakis, M. Heindl, C. Houghton, D. Jaroslawski, S. Konstantinou, I. Laflotte, A. Lath, J. Martins, B. Rand, J. Reichert, P. Saha, S. Salur, S. Schnetzer, S. Somalwar, R. Stone, S. A. Thayil, S. Thomas, J. Vora, D. Ally, A. G. Delannoy, S. Fiorendi, J. Harris, S. Higginbotham, T. Holmes, A. R. Kanuganti, N. Karunarathna, J. Lawless, L. Lee, E. Nibigira, B. Skipworth, S. Spanier, D. Aebi, M. Ahmad, T. Akhter, K. Androsov, A. Bolshov, O. Bouhali, A. Cagnotta, V. D’Amante, R. Eusebi, P. Flanagan, J. Gilmore, Y. Guo, T. Kamon, S. Luo, R. Mueller, A. Safonov, N. Akchurin, J. Damgov, Y. Feng, N. Gogate, Y. Kazhykarim, K. Lamichhane, S. W. Lee, C. Madrid, A. Mankel, T. Peltola, I. Volobouev, E. Appelt, Y. Chen, S. Greene, A. Gurrola, W. Johns, R. Kunnawalkam Elayavalli, A. Melo, D. Rathjens, F. Romeo, P. Sheldon, S. Tuo, J. Velkovska, J. Viinikainen, J. Zhang, B. Cardwell, H. Chung, B. Cox, J. Hakala, R. Hirosky, M. Jose, A. Ledovskoy, C. Mantilla, C. Neu, C. Ramón Álvarez, S. Bhattacharya, P. E. Karchin, A. Aravind, S. Banerjee, K. Black, T. Bose, E. Chavez, S. Dasu, P. Everaerts, C. Galloni, H. He, M. Herndon, A. Herve, C. K. Koraka, S. Lomte, R. Loveless, A. Mallampalli, A. Mohammadi, S. Mondal, T. Nelson, G. Parida, L. Pétré, D. Pinna, A. Savin, V. Shang, V. Sharma, W. H. Smith, D. Teague, H. F. Tsoi, W. Vetens, A. Warden, S. Afanasiev, V. Alexakhin, Yu. Andreev, T. Aushev, D. Budkouski, R. Chistov, M. Danilov, T. Dimova, A. Ershov, S. Gninenko, I. Gorbunov, A. Gribushin, A. Kamenev, V. Karjavine, M. Kirsanov, V. Klyukhin, O. Kodolova, V. Korenkov, A. Kozyrev, N. Krasnikov, A. Lanev, A. Malakhov, V. Matveev, A. Nikitenko, V. Palichik, V. Perelygin, S. Petrushanko, S. Polikarpov, O. Radchenko, M. Savina, V. Shalaev, S. Shmatov, S. Shulha, Y. Skovpen, V. Smirnov, O. Teryaev, I. Tlisova, A. Toropin, N. Voytishin, B. S. Yuldashev, A. Zarubin, I. Zhizhin, L. Dudko, K. Ivanov, V. Kim, V. Murzin, V. Oreshkin, D. Sosnov, E. Boos, V. Bunichev, M. Dubinin, V. Savrin, A. Snigirev

**Affiliations:** 1 Yerevan Physics Institute, Yerevan, Armenia; 2 Institut für Hochenergiephysik, Vienna, Austria; 3 Universiteit Antwerpen, Antwerpen, Belgium; 4 Vrije Universiteit Brussel, Brussels, Belgium; 5 Ghent University, Ghent, Belgium; 6 Université Libre de Bruxelles, Brusells, Belgium; 7 Université Catholique de Louvain, Louvain-la-Neuve, Belgium; 8 Centro Brasileiro de Pesquisas Fisicas, Rio de Janeiro, Brazil; 9 Universidade do Estado do Rio de Janeiro, Rio de Janeiro, Brazil; 10 Universidade Estadual Paulista, Universidade Federal do ABC, São Paulo, Brazil; 11 Institute for Nuclear Research and Nuclear Energy, Bulgarian Academy of Sciences, Sofia, Bulgaria; 12 University of Sofia, Sofia, Bulgaria; 13 Instituto De Alta Investigación, Universidad de Tarapacá, Arica, Chile; 14 Universidad Técnica Federico Santa María, Valparaiso, Chile; 15 Beihang University, Beijing, China; 16 Department of Physics, Tsinghua University, Beijing, China; 17 Institute of High Energy Physics, Beijing, China; 18 State Key Laboratory of Nuclear Physics and Technology, Peking University, Beijing, China; 19 Guangdong Provincial Key Laboratory of Nuclear Science and Guangdong-Hong Kong Joint Laboratory of Quantum Matter, South China Normal University, Guangzhou, China; 20 Sun Yat-Sen University, Guangzhou, China; 21 University of Science and Technology of China, Hefei, China; 22 Nanjing Normal University, Nanjing, China; 23 The University of Iowa, Iowa City, IA USA; 24 Institute of Modern Physics and Key Laboratory of Nuclear Physics and Ion-beam Application (MOE) - Fudan University, Shanghai, China; 25 Zhejiang University, Hangzhou, China; 26 Universidad de Los Andes, Bogota, Colombia; 27 Universidad de Antioquia, Medellin, Colombia; 28 Faculty of Electrical Engineering, Mechanical Engineering and Naval Architecture University of Split, Split, Croatia; 29 Faculty of Science, University of Split, Split, Croatia; 30 Institute Rudjer Boskovic, Zagreb, Croatia; 31 University of Cyprus, Nicosia, Cyprus; 32 Charles University, Prague, Czech Republic; 33 Escuela Politecnica Nacional, Quito, Ecuador; 34 Universidad San Francisco de Quito, Quito, Ecuador; 35 Academy of Scientific Research and Technology of the Arab Republic of Egypt, Egyptian Network of High Energy Physics, Cairo, Egypt; 36 Center for High Energy Physics (CHEP-FU), Fayoum University, Faiyum, Egypt; 37 National Institute of Chemical Physics and Biophysics, Tallinn, Estonia; 38 Department of Physics, University of Helsinki, Helsinki, Finland; 39 Helsinki Institute of Physics, Helsinki, Finland; 40 Lappeenranta-Lahti University of Technology, Lappeenranta, Finland; 41 IRFU, CEA, Université Paris-Saclay, Gif-sur-Yvette, France; 42 Purdue University, West Lafayette IN, USA; 43 Laboratoire Leprince-Ringuet, CNRS/IN2P3, École Polytechnique, Institut Polytechnique de Paris, Palaiseau, France; 44 Université de Strasbourg, CNRS IPHC UMR7178, Strasbourg, France; 45 Centre de Calcul de l’Institut National de Physique Nucleaire et de Physique des Particules, CNRS/IN2P3, Villeurbanne, France; 46 Institut de Physique des2Infinis de Lyon (IP2I), Villeurbanne, France; 47 Georgian Technical University, Tbilisi, Georgia; 48 I. Physikalisches Institut, RWTH Aachen University, Aachen, Germany; 49 III. Physikalisches Institut A, RWTH Aachen University, Aachen, Germany; 50 III. Physikalisches Institut B, RWTH Aachen University, Aachen, Germany; 51 Deutsches Elektronen-Synchrotron, Hamburg, Germany; 52 University of Hamburg, Hamburg, Germany; 53 Karlsruher Institut fuer Technologie, Karlsruhe, Germany; 54 CERN, European Organization for Nuclear Research, Geneva, Switzerland; 55 Institute of Nuclear and Particle Physics (INPP), NCSR Demokritos, Aghia Paraskevi, Greece; 56 National and Kapodistrian University of Athens, Athens, Greece; 57 National Technical University of Athens, Athens, Greece; 58 University of Ioánnina, Ioannina, Greece; 59 HUN-REN Wigner Research Centre for Physics, Budapest, Hungary; 60 HUN-REN Institute of Nuclear Research - ATOMKI, Debrecen, Hungary; 61 MTA-ELTE Lendület CMS Particle and Nuclear Physics Group, Eötvös Loránd University, Budapest, Hungary; 62 Faculty of Informatics, University of Debrecen, Debrecen, Hungary; 63 Karoly Robert Campus, MATE Institute of Technology, Gyongyos, Hungary; 64 The University of Kansas, Lawrence, KS USA; 65 Panjab University, Chandigarh, India; 66 University of Delhi, New Delhi, India; 67 University of Hyderabad, Hyderabad, India; 68 Indian Institute of Technology Kanpur, Kanpur, India; 69 Saha Institute of Nuclear Physics, Homi Bhabha National Institute, Kolkata, India; 70 Indian Institute of Technology Madras, Chennai, India; 71 IISER Mohali, Mohali, India; 72 Tata Institute of Fundamental Research-A, Mumbai, India; 73 Tata Institute of Fundamental Research-B, Mumbai, India; 74 National Institute of Science Education and Research, Bhubaneswar, India; 75 Indian Institute of Science Education and Research (IISER), Pune, India; 76 Indian Institute of Technology Hyderabad, Telangana, India; 77 Isfahan University of Technology, Isfahan, Iran; 78 Institute for Research in Fundamental Sciences (IPM), Tehran, Iran; 79 University College Dublin, Dublin, Ireland; 80 INFN Sezione di Bari, Bari, Italy; 81 Università di Bari, Bari, Italy; 82 Politecnico di Bari, Bari, Italy; 83 INFN Sezione di Bologna, Bologna, Italy; 84 Università di Bologna, Bologna, Italy; 85 INFN Sezione di Catania, Catania, Italy; 86 Università di Catania, Catania, Italy; 87 INFN Sezione di Firenze, Firenze, Italy; 88 Università di Firenze, Firenze, Italy; 89 INFN Laboratori Nazionali di Frascati, Frascati, Italy; 90 INFN Sezione di Genova, Genova, Italy; 91 Università di Genova, Genova, Italy; 92 INFN Sezione di Milano-Bicocca, Milano, Italy; 93 Università di Milano-Bicocca, Milano, Italy; 94 INFN Sezione di Napoli, Napoli, Italy; 95 Università di Napoli ‘Federico II’, Napoli, Italy; 96 Università della Basilicata, Potenza, Italy; 97 Scuola Superiore Meridionale (SSM), Napoli, Italy; 98 INFN Sezione di Padova, Padova, Italy; 99 Fermi National Accelerator Laboratory, Batavia, IL USA; 100 Università di Padova, Padova, Italy; 101 INFN Sezione di Pavia, Pavia, Italy; 102 Università di Pavia, Pavia, Italy; 103 INFN Sezione di Perugia, Perugia, Italy; 104 Università di Perugia, Perugia, Italy; 105 INFN Sezione di Pisa, Pisa, Italy; 106 Università di Pisa, Pisa, Italy; 107 Scuola Normale Superiore di Pisa, Pisa, Italy; 108 Università di Siena, Siena, Italy; 109 INFN Sezione di Roma, Roma, Italy; 110 Sapienza Università di Roma, Roma, Italy; 111 INFN Sezione di Torino, Torino, Italy; 112 Università di Torino, Torino, Italy; 113 Università del Piemonte Orientale, Novara, Italy; 114 INFN Sezione di Trieste, Trieste, Italy; 115 Università di Trieste, Trieste, Italy; 116 Kyungpook National University, Daegu, Korea; 117 Department of Mathematics and Physics, Gangneung-Wonju National University, Gangneung, Korea; 118 Institute for Universe and Elementary Particles, Chonnam National University, Kwangju, Korea; 119 Hanyang University, Seoul, Korea; 120 Korea University, Seoul, Korea; 121 Department of Physics, Kyung Hee University, Seoul, Korea; 122 Sejong University, Seoul, Korea; 123 Seoul National University, Seoul, Korea; 124 University of Seoul, Seoul, Korea; 125 Department of Physics, Yonsei University, Seoul, Korea; 126 Sungkyunkwan University, Suwon, Korea; 127 College of Engineering and Technology, American University of the Middle East (AUM), Dasman, Kuwait; 128 College of Science, Department of Physics, Kuwait University, Safat, Kuwait; 129 Riga Technical University, Riga, Latvia; 130 University of Latvia (LU), Riga, Latvia; 131 Vilnius University, Vilnius, Lithuania; 132 National Centre for Particle Physics, Universiti Malaya, Kuala Lumpur, Malaysia; 133 Universidad de Sonora (UNISON), Hermosillo, Mexico; 134 Centro de Investigacion y de Estudios Avanzados del IPN, Mexico City, Mexico; 135 Universidad Iberoamericana, Mexico City, Mexico; 136 Benemerita Universidad Autonoma de Puebla, Puebla, Mexico; 137 University of Montenegro, Podgorica, Montenegro; 138 University of Canterbury, Christchurch, New Zealand; 139 National Centre for Physics, Quaid-I-Azam University, Islamabad, Pakistan; 140 AGH University of Krakow, Krakow, Poland; 141 National Centre for Nuclear Research, Swierk, Poland; 142 Institute of Experimental Physics, Faculty of Physics, University of Warsaw, Warsaw, Poland; 143 Warsaw University of Technology, Warsaw, Poland; 144 Laboratório de Instrumentação e Física Experimental de Partículas, Lisbon, Portugal; 145 Faculty of Physics, University of Belgrade, Belgrade, Serbia; 146 VINCA Institute of Nuclear Sciences, University of Belgrade, Belgrade, Serbia; 147 Centro de Investigaciones Energéticas Medioambientales y Tecnológicas (CIEMAT), Madrid, Spain; 148 Universidad Autónoma de Madrid, Madrid, Spain; 149 Instituto Universitario de Ciencias y Tecnologías Espaciales de Asturias (ICTEA), Universidad de Oviedo, Oviedo, Spain; 150 Instituto de Física de Cantabria (IFCA), CSIC-Universidad de Cantabria, Santander, Spain; 151 University of Colombo, Colombo, Sri Lanka; 152 Department of Physics, University of Ruhuna, Matara, Sri Lanka; 153 PSI Center for Neutron and Muon Sciences, Villigen, Switzerland; 154 Universität Zürich, Zurich, Switzerland; 155 Institute for Particle Physics and Astrophysics (IPA), ETH Zurich, Zurich, Switzerland; 156 National Central University, Chung-Li, Taiwan; 157 National Taiwan University (NTU), Taipei, Taiwan; 158 High Energy Physics Research Unit, Department of Physics, Faculty of Science, Chulalongkorn University, Bangkok, Thailand; 159 Tunis El Manar University, Tunis, Tunisia; 160 Physics Department, Science and Art Faculty, Ҫukurova University, Adana, Turkey; 161 Physics Department, Middle East Technical University, Ankara, Turkey; 162 Bogazici University, Istanbul, Turkey; 163 Istanbul Technical University, Istanbul, Turkey; 164 Istanbul University, Istanbul, Turkey; 165 Yildiz Technical University, Istanbul, Turkey; 166 Institute for Scintillation Materials, National Academy of Science of Ukraine, Kharkiv, Ukraine; 167 National Science Centre Kharkiv Institute of Physics and Technology, Kharkiv, Ukraine; 168 University of Bristol, Bristol, UK; 169 Rutherford Appleton Laboratory, Didcot, UK; 170 Imperial College, London, UK; 171 Brunel University, Uxbridge, UK; 172 Baylor University, Waco, TX USA; 173 Bethel University, St Paul, MN USA; 174 Catholic University of America, Washington, DC USA; 175 The University of Alabama, Tuscaloosa, AL USA; 176 Boston University, Boston, MA USA; 177 Brown University, Providence, RI USA; 178 University of California Davis, Davis, CA USA; 179 University of California Los Angeles, Los Angeles CA, USA; 180 University of California Riverside, Riverside, CA USA; 181 University of California San Diego, La Jolla, Ca USA; 182 Department of Physics, University of California Santa Barbara, Santa Barbara, CA USA; 183 California Institute of Technology, Pasadena, CA USA; 184 Carnegie Mellon University, Pittsburgh, PA USA; 185 University of Colorado Boulder, Boulder, CO USA; 186 Cornell University, thaca NY, USA; 187 University of Florida, Gainesville, FL USA; 188 Florida State University, Tallahassee, FL USA; 189 Florida Institute of Technology, Melbourne, FL USA; 190 University of Illinois Chicago, Chicago, IL USA; 191 Johns Hopkins University, Baltimore, MD USA; 192 Kansas State University, Manhattan, KS USA; 193 University of Maryland, College Park, MD USA; 194 Massachusetts Institute of Technology, Cambridge, MA USA; 195 University of Minnesota, Minneapolis, MN USA; 196 University of Nebraska–Lincoln, Lincoln, NE USA; 197 State University of New York at Buffalo, Buffalo, NY USA; 198 Northeastern University, Boston, MA USA; 199 Northwestern University, Evanston, IL USA; 200 University of Notre Dame, Notre Dame IN, USA; 201 The Ohio State University, Columbus OH, USA; 202 Princeton University, Princeton, NJ USA; 203 University of Puerto Rico, Mayaguez, PR USA; 204 Purdue University Northwest, Hammond IN, USA; 205 Rice University, Houston, TX USA; 206 University of Rochester, Rochester NY, USA; 207 Rutgers, The State University of New Jersey, Piscataway, NJ USA; 208 University of Tennessee, Knoxville, TN USA; 209 Texas A&M University, College Station, TX USA; 210 Texas Tech University, Lubbock, TX USA; 211 Vanderbilt University, Nashville, TN USA; 212 University of Virginia, Charlottesville, VA USA; 213 Wayne State University, Detroit, MI USA; 214 University of Wisconsin–Madison, Madison, WI USA; 215 An institute or international laboratory covered by a cooperation agreement with CERN, Geneva, Switzerland; 216 An institute formerly covered by a cooperation agreement with CERN, Geneva, Switzerland

**Keywords:** Particle physics, Physics

## Abstract

The traditional quark model^1,2^ accounts for the existence of baryons, such as protons and neutrons, which consist of three quarks, as well as mesons, composed of a quark–antiquark pair. Only recently has substantial evidence started to accumulate for exotic states composed of four or five quarks and antiquarks^3^. The exact nature of their internal structure remains uncertain^4–29^. Here we report the first measurement of quantum numbers of the recently discovered family of three all-charm tetraquarks^30–32^, using data collected by the CMS experiment at the Large Hadron Collider from 2016 to 2018 (refs. ^33,34^). The angular analysis techniques developed for the discovery and characterization of the Higgs boson^35–37^ have been applied to the new exotic states. Here we show that the quantum numbers for parity *P* and charge conjugation *C* symmetries are found to be +1. The spin *J* of these exotic states is determined to be consistent with 2*ħ*, while 0*ħ* and 1*ħ* are excluded at 95% and 99% confidence levels, respectively. The *J*^*PC*^ = 2^++^ assignment implies particular configurations of constituent spins and orbital angular momenta, which constrain the possible internal structure of these tetraquarks.

## Abstract (web summary)

The CMS Collaboration reports the measurement of the spin, parity, and charge conjugation properties of all-charm tetraquarks, exotic fleeting particles formed in proton–proton collisions at the Large Hadron Collider.

## Main

In 1964, Gell-Mann^1^ and Zweig^2^ independently proposed that hadrons, such as protons and neutrons, are made up of elementary particles called quarks (q). They suggested that mesons and baryons consisted of quark–antiquark pairs (${\rm{q}}\bar{{\rm{q}}}$) and quark triplets (qqq), respectively. At the time, only three quark types were proposed: the up (u) and down (d), which form protons (uud) and neutrons (udd) and the strange quarks. The discovery of the J/Ψ meson in 1974 (refs. ^38,39^) was soon recognized as evidence of a fourth quark type, charm (c), bound with its corresponding antiquark. Gell-Mann and Zweig also pointed out that the symmetry principles underlying their model allowed for ‘exotic’ configurations, such as tetraquark and pentaquark systems. Despite numerous searches, the existence of these particles remained uncertain by the end of the last century^40^.

The study of exotic hadrons advanced when access to large samples of hadrons containing heavy c and b quarks became available, highlighted by the 2003 discovery of the new particle X(3872) in J/Ψ π^+^π^−^ decays^41^. This particle has been interpreted as a tetraquark candidate with content ${\rm{u}}\mathrm{c}\bar{{\rm{u}}}\bar{{\rm{c}}}$ and recently classified as *χ*_c1_(3872) (ref. ^3^), where the number denotes its mass in MeV (megaelectronvolts) and we adopt natural units by setting *c*  = 1 and *ħ* = 1. The discovery of several other candidates for tetraquarks and pentaquarks followed, including a ${\rm{u}}{\rm{c}}\bar{{\rm{d}}}\bar{{\rm{c}}}$ candidate Z_c_(3900)^+^ → J/Ψπ^+^ (refs. ^42,43^), also classified as ${{\rm{T}}}_{{\rm{c}}\bar{{\rm{c}}}1}{(3900)}^{+}$ (ref. ^3^). However, the true nature of their structure continues to be widely debated^4–29^. Most interpretations fall into two categories: tightly bound states of two quarks and two antiquarks, similar to how quarks are bound within protons and neutrons, or loosely bound molecules composed of two mesons, similar to how protons and neutrons compose an atomic nucleus.

**Fig. 1:**
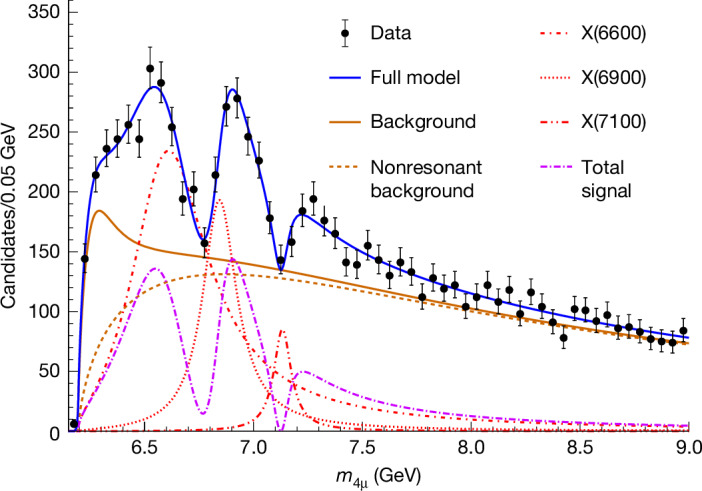
Candidates for all-charm tetraquarks. The J/Ψ J/Ψ → μ^+^μ^−^μ^+^μ^−^ invariant mass *m*_4μ_ spectrum shows the three exotic states, X(6600), X(6900) and X(7100). Parameterizations of these states are shown both individually and as a combined signal that includes quantum-mechanical interference (denoted by Total signal). The full model^32^ incorporates both signal and background components, with the background originating from di-J/Ψ production, including contributions from nonresonant production and an enhancement near the kinematic threshold of 6.2 GeV.

The next breakthrough in understanding exotic hadrons may come from studying those composed entirely of heavy quarks. The LHCb, ATLAS and CMS Collaborations observed a state X(6900) (refs. ^30–32^), also referred to as ${{\rm{T}}}_{{\rm{c}}{\rm{c}}\bar{{\rm{c}}}\bar{{\rm{c}}}}{(6900)}^{0}$ (ref. ^3^), in the J/Ψ J/Ψ final state. The CMS Collaboration also reported two additional states, X(6600) and X(7100). The triple-peaking structure in the di-J/Ψ invariant mass distribution observed by CMS^32^ is shown in Fig. 1 and is best described by a quantum-mechanical interference of three amplitudes representing ${\rm{c}}{\rm{c}}\bar{{\rm{c}}}\bar{{\rm{c}}}$ states. The interference pattern among the three resonances, along with the mass spacings that follow a radial Regge trajectory^29^, suggests that these three X particles form a family of states with identical properties, differing only in the radial excitations of their wavefunctions. These properties, which include the spin and symmetry quantum numbers, provide insights into the internal structure of these exotic hadrons and are the primary focus of the experimental study reported here. The corresponding tabulated results are available in the HEPData record for this analysis^44^.

## The spin and symmetry properties

Spin is a type of intrinsic angular momentum carried by particles. For composite particles, the total spin *J* represents the total angular momentum of the system, determined by the combination of the spins *S* and orbital angular momenta *L* of the elementary constituent particles. The quantum number for parity (*P*) symmetry describes how a system behaves under a spatial inversion or, equivalently, a mirror reflection. The charge conjugation (*C*) symmetry describes how a system transforms when every particle is replaced by its corresponding antiparticle. The *P* = ±1 and *C* = ±1 values indicate whether the wavefunction of the state changes sign under the respective transformation.

**Fig. 2:**
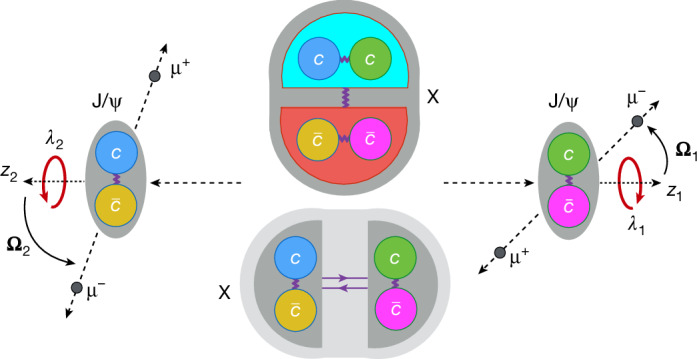
Internal structure models for the particle X. The particle X, composed of ${\rm{c}}{\rm{c}}\bar{{\rm{c}}}\bar{{\rm{c}}}$, is shown at rest. Two models of the internal structure of X are presented: a tightly bound tetraquark (top) and a loosely bound molecule of two mesons (bottom). The colours assigned to individual quarks or quark pairs denote possible colour charge assignments in strong interactions, in which attractive forces are mediated by gluon exchange (shown as wavy lines) and meson exchange (shown as a solid pair of arrows). The X decays into two J/Ψ mesons with spin projections *λ*_*i*_ along their respective directions of motion; each meson then decays into a μ^+^μ^−^ pair. The polar and azimuthal angles **Ω**_*i*_ = (*θ*_*i*_, *Φ*_*i*_) describe the direction of the μ^−^ relative to the *z*_*i*_-axis, which is defined to point opposite to the X-direction in the centre-of-mass frame of the corresponding J/Ψ meson, for *i* = 1 and 2.

The quarks and antiquarks have a spin of *S* = 1/2, the fundamental quantum of spin, and they serve as the building blocks of the tetraquark state shown in Fig. 2. The quarks inside a hadron can carry three different colour charges, associated with the strong interaction, conventionally referred to as blue, green and red. Antiquarks carry corresponding anticolour charges (antiblue, antigreen and antired), which are shown as yellow, magenta and cyan, respectively, in Fig. 2. Quarks and antiquarks are held together through the exchange of gluons, which are the mediators of the strong interaction. The hadron as a whole is colour-charge-neutral.

A tightly bound tetraquark state X is shown in Fig. 2 (top). In the ground state with zero orbital angular momentum, two identical charm quarks form an antisymmetric colour state for attraction, leading to symmetric spatial and spin states with total spin 1 (ref. ^25^). The two charm antiquarks do the same. Each pair carries a colour charge as well, and the two pairs attract each other, forming a strongly bound tetraquark state that is colour-charge-neutral, similar to a quark–antiquark bound state in a meson. The orbital angular momentum *L* between the quark pair and the antiquark pair can take non-negative integer values. The corresponding parity of the system is then given by *P* = (−1)^*L*^, which arises from the behaviour of the spherical harmonics under spatial inversion.

The lowest and most probable energy state with *L* = 0 is spatially symmetric, *P* = +1. The spins of the two systems combine in a symmetric configuration to yield a total spin *J* = 0 or 2 with *C* = +1, or in an antisymmetric configuration to give *J* = 1 with *C* = −1. For the cc and $\bar{{\rm{c}}}\bar{{\rm{c}}}$ system, charge conjugation is equivalent to exchanging the pairs, resulting in the associated symmetry. As we will demonstrate later, the *C* = −1 configuration is ruled out and will, therefore, not be considered further. For an antisymmetric spatial state with *L* = 1 and *P* = −1, the spins combine to 1 in an antisymmetric configuration and with *C* = +1, resulting in possible total spins *J* = 0, 1 or 2. States with *L* = 2 are also possible, resulting in *P* = +1 and allowing *J* values up to 4 when the spins combine to 0 or 2. However, the high orbital angular momentum requires additional energy, making these states less likely.

An alternative model, shown in Fig. 2 (bottom), is a loosely bound molecule of two ${\rm{c}}\bar{{\rm{c}}}$ mesons. The lowest-energy configuration corresponds to an orbital angular momentum *L* = 0 between the two mesons, resulting in *P* = +1. A key distinction is that, unlike in a tightly bound tetraquark, the two constituent ${\rm{c}}\bar{{\rm{c}}}$ mesons are not restricted to form spin-1 states. Consequently, lower total spin values such as *J* = 0 or 1 are more likely, although higher spin states cannot be excluded. Another difference is the weaker interaction between the ${\rm{c}}\bar{{\rm{c}}}$ mesons. Similar to how a deuteron is a bound state of a proton and a neutron, the two colour-charge-neutral systems are bound through the exchange of a meson by the Yukawa interaction^45^. However, unlike the deuteron, in an all-charm tetraquark molecule, the exchanged meson must contain charm quarks. A heavier exchange meson substantially suppresses the Yukawa interaction, making the formation of bound states less likely. However, alternative empirical models for these interactions have also been explored^9–12^.

The three X states under investigation have invariant masses ranging between 6.2 GeV and 8.0 GeV (gigaelectronvolts), as shown in Fig. 1, mean lifetimes between 10^−24^ s and 10^−23^ s (ref. ^32^), and they decay into either two J/Ψ mesons, or potentially several other, yet unobserved, final states. The J/Ψ meson has a mass of 3.1 GeV, spin 1, a mean lifetime of approximately 7 × 10^−21^ s, and in 6% of the cases, the ${\rm{c}}\bar{{\rm{c}}}$ pair in the J/Ψ annihilates into a μ^+^μ^−^ pair^3^, which is ideal for the detection of the final states and for performing an angular analysis.

## Angular distributions

The X quantum numbers can be inferred from the polarizations of the J/Ψ mesons, which, in turn, result in specific angular distributions of the decay muons. Figure 2 defines *λ*_1_ and *λ*_2_ as the spin projections of the two J/Ψ mesons along their respective directions of motion. Both *λ*_1_ and *λ*_2_ can take values of −1, 0 or +1. Let ${A}_{{\lambda }_{1}{\lambda }_{2}}$ be the quantum-mechanical amplitude that describes the decay of X into two mesons with spin projections *λ*_1_ and *λ*_2_. The nine values of ${| {A}_{{\lambda }_{1}{\lambda }_{2}}| }^{2}$ can be interpreted as relative probabilities for specific polarization states of the two J/Ψ mesons.

The angular distributions in the decay X → J/Ψ J/Ψ → (μ^+^μ^−^)(μ^+^μ^−^) can be expressed as a function of three angular observables *θ*_1_, *θ*_2_ and *Φ* = (*Φ*_1_ + *Φ*_2_) as shown in Fig. 2 and further explained in the section ‘Angular observables’. Here, *θ*_1_ and *θ*_2_ are the helicity angles of the two J/Ψ mesons, and *Φ* represents the angle between their decay planes. The nine amplitudes ${A}_{{\lambda }_{1}{\lambda }_{2}}$ appear in the coefficients of the expression. The analytical and numerical calculations of the angular distributions are detailed in refs. ^35,46^.

We base the prediction of ${A}_{{\lambda }_{1}{\lambda }_{2}}$ on symmetry considerations^46^. Angular momentum conservation implies that |*λ*_1_ − *λ*_2_| ≤ *J*. For two identical J/Ψ particles, the relation ${A}_{{\lambda }_{1}{\lambda }_{2}}={(-1)}^{J}{A}_{{\lambda }_{2}{\lambda }_{1}}$ must hold. Finally, assuming that the X has definite *P* and *C* quantum numbers, which are conserved in the strong decays, we conclude that ${A}_{{\lambda }_{1}{\lambda }_{2}}=P{(-1)}^{J}{A}_{(-{\lambda }_{2})(-{\lambda }_{1})}$ and the charge conjugation *C* = +1. The latter is due to the *C* = +1 of the J/Ψ J/Ψ final state. This results in six possible assignments for the *J*^*PC*^ quantum numbers with the corresponding contributions from the amplitudes shown in Table 1. States *J*^*P*+^ with *J* ≥ 3 would exhibit amplitudes and angular distributions similar to those of 2^*P*+^, as inferred from ref. ^46^.

**Table 1:** Quantum numbers

*J*^*PC*^	Models ${{\boldsymbol{J}}}_{{\boldsymbol{i}}}^{{\boldsymbol{P}}}$	Contributing amplitudes
0^−+^	0^−^	*A*_++_ = −*A*_−−_
0^++^	${0}_{{\rm{m}}}^{+}$	*A*_00_ and *A*_++_ = *A*_−−_
	${0}_{{\rm{h}}}^{+}$	*A*_++_ = *A*_−−_ and *A*_00_
1^−+^	1^−^	*A*_+0_ = −*A*_0+_ = *A*_−0_ = −*A*_0−_
1^++^	1^+^	*A*_+0_ = −*A*_0+_ = −*A*_−0_ = *A*_0−_
2^−+^	${2}_{{\rm{m}}}^{-}$	*A*_++_ = −*A*_−−_
	${2}_{{\rm{h}}}^{-}$	*A*_+0_ = *A*_0+_ = −*A*_−0_ = −*A*_0−_
2^++^	${2}_{{\rm{m}}}^{+}$	*A*_+0_ = *A*_0+_ = *A*_−0_ = *A*_0−_, *A*_+−_ = *A*_−+_, *A*_00_ and *A*_++_ = *A*_−−_

The possible assignments of quantum numbers *J*^*PC*^, the ${J}_{i}^{P}$ models considered, and the contributing amplitudes in the decay X → J/Ψ J/Ψ are presented.

As shown in Table 1, in the scenarios of 0^−+^, 1^−+^ and 1^++^, the relative contributions of all amplitudes ${A}_{{\lambda }_{1}{\lambda }_{2}}$ are predetermined. In the scenarios of 0^++^ and 2^−+^, the relative contributions of the two different sets of amplitudes, associated with different models ${J}_{i}^{P}$ listed in Table 1, are not known a priori. The 2^++^ scenario has four contributions with an undetermined relationship. When pure symmetry alone does not provide a determination, the dynamical properties of the interactions provide additional constraints that relate the contributions. Assuming the resonances differ only by radial excitations of their wavefunctions, we expect all three to exhibit the same structure in their decay interactions. This structure and the corresponding models are defined and motivated in the section ‘Modelling of hadron properties’.

The ${0}_{{\rm{m}}}^{+}$ model describes the simplest possible structure for the interaction of a spin-0 particle decaying into two spin-1 particles, with the subscript ‘m’ indicating its minimal nature. By contrast, the ${0}_{{\rm{h}}}^{+}$ model corresponds to a more complex interaction structure, as denoted by the subscript ‘h’ for higher complexity^35^. The ${2}_{{\rm{m}}}^{-}$ and ${2}_{{\rm{h}}}^{-}$ models correspond to *A*_++_ = −*A*_−−_ and *A*_+0_ = *A*_0+_ = −*A*_−0_ = −*A*_0−_, and their decay angular distributions are indistinguishable from those of 0^−^ and 1^−^ models, respectively. Quantum-mechanical interference between models ${0}_{{\rm{m}}}^{+}$ and ${0}_{{\rm{h}}}^{+}$, or ${2}_{{\rm{m}}}^{-}$ and ${2}_{{\rm{h}}}^{-}$, is also taken into account in the data analysis. The ${2}_{{\rm{m}}}^{+}$ model, as defined in ref. ^35^, corresponds to the minimal structure of interactions of a spin-2 particle in its decay. It is used to represent the 2^++^ quantum numbers with a distinct contribution from *A*_+−_ = *A*_−+_, which is unique to 2^++^. Moreover, seven other amplitudes contribute simultaneously, another feature unique to 2^++^. Other possible 2^++^ models, beyond the minimal structure, may exhibit angular distributions similar to those of the 0^++^ or 1^++^ states and are not tested separately.

The reasoning outlined above leads to the predicted probability distributions ${{\mathcal{P}}}_{i}({\theta }_{1},{\theta }_{2},\varPhi ,{m}_{4{\rm{\mu }}})$ for the eight individual ${J}_{i}^{P}$ models listed in Table 1, as well as their possible mixtures, at any mass value *m*_4μ_ for the particle decay X → J/Ψ J/Ψ. Our analysis aims to identify the model that best matches the experimental data.

## Experimental data

To create new particles, proton beams are accelerated to a combined energy of 13 TeV (teraelectronvolts) in opposite directions within the Large Hadron Collider (LHC)^47^, an international project hosted by CERN, and collided head-on. Protons are baryons with a mass of 0.94 GeV, composed of three valence quarks. However, approximately half of their momentum is carried by gluons, which bind the quarks together. Furthermore, sea quark–antiquark pairs emerge and carry roughly one-fifth of the momentum of the proton. As a result, new particles can be created through collisions between gluons, quarks and antiquarks (collectively referred to as partons) within the protons. The data presented in this paper were collected from 2016 to 2018, with a total integrated luminosity of 135 fb^−1^ (inverse femtobarns), which represents approximately 1.5 × 10^16^ proton–proton (pp) collisions.

Once produced, the X particles immediately decay within the CMS detector^33,34^, a large general-purpose international experiment at the LHC. Its essential component is a superconducting solenoid with an internal diameter of 6 m, which can generate a magnetic field of 3.8 T (tesla). Within the volume of the solenoid, there are a silicon pixel and strip tracker, a lead tungstate crystal electromagnetic calorimeter and a brass and scintillator hadron calorimeter, each consisting of a barrel and two endcap sections. Muons are identified using gas-ionization chambers embedded in the steel flux-return yoke outside the solenoid. In this analysis, the primary measurements of muon momentum vectors are obtained from the curvature and direction of their paths in the silicon tracker. A fast, real-time data recording decision is made using a two-level trigger system^48^, which reduces the recorded data rate to approximately 1 kHz (kilohertz).

To select events of interest, the trigger criteria include having three muons each with a minimum muon momentum transverse to the beam (*p*_T_) of 3 GeV. The procedure for the offline selection of X candidates is described in ref. ^32^: there must be at least four muons, each with *p*_T_ > 2 GeV; μ^+^μ^−^ pairs must originate from a common vertex, have a mass consistent with a J/Ψ, and the muons from the highest-*p*_T_ J/Ψ satisfy *p*_T_ > 3.5 GeV. A fit is applied to muon pairs, using the J/Ψ meson mass and the common production vertex as constraints, to improve the *m*_4μ_ resolution. A total of 8,651 candidate events with two J/Ψ mesons have been recorded with a combined mass in the range of 6.2–9.0 GeV, as shown in Fig. 1, with approximately a quarter expected to correspond to the three X states, and the remainder mostly originating from background J/Ψ pair production. These candidates are the same as those in ref. ^32^. For each X candidate, we record the momentum vectors of all four muons for the analysis that follows.

## Angular analysis

The objective of the analysis is to compare the angular distributions in the decay of X particles observed in the experiment with the predicted distributions for various *J*^*PC*^ models and select the model that is consistent with the data. To prevent a potential bias, the observed distributions were hidden until all aspects of the analysis were finalized. The predicted distributions ${{\mathcal{P}}}_{i}({\theta }_{1},{\theta }_{2},\varPhi ,{m}_{4{\rm{\mu }}})$ are precisely known for the muons originating from the decay, but the observed distributions are modified by detector effects. This is because the detection probabilities of the reconstructed muons depend on their momenta and directions, which have complex correlations with the angles. Moreover, the momenta and directions themselves are subject to uncertainties. To account for these effects and to accurately model the background, a comprehensive computer simulation of pp collisions is performed using Monte Carlo (MC) techniques. The simulation proceeds in four stages.

In the first stage, the JHUGEN program, v.7.5.7 (refs. ^35,46^), originally designed to model variations in the properties of the Higgs boson, is used to simulate the process pp → X → J/Ψ J/Ψ → μ^+^μ^−^μ^+^μ^−^. Two models of X production are explored^9–12^: direct production through parton annihilation within protons and fragmentation of a secondary gluon or quark generated in collisions. In the case of *J* ≥ 1, the first model can lead to a preferred spin polarization of X along the beam axis, whereas the second model may result in polarization along the direction of motion of the X particle. JHUGEN enables the modelling of either polarization or the simulation of an unpolarized state through the application of event weights. Polarization variations are used to estimate uncertainties, with the unpolarized case assumed by default.

The three stages of the simulation that follow are shared with thousands of other processes analysed by the CMS Collaboration and have, therefore, been extensively tuned to ensure a good agreement with the experimental data. The simulation of the remaining particles appearing in proton collisions and surrounding the four muons is performed by the pythia program, v.8.240 (ref. ^49^). The simulation takes into account the effects of extra pp collisions that happen at the same time or close in time to the main collision. The generated events are further processed through a dedicated CMS detector simulation, based on the Geant4 program^50^, which models the detector response. Finally, the simulated events undergo the complete detector reconstruction process, using software and algorithms identical to those used to analyse the experimental data.

The nonresonant J/Ψ J/Ψ background is simulated with pythia in the first stage and covers cases in which the two mesons are produced either in the same or in different parton collisions in a single pp interaction. The simulated angular distributions were found to be compatible with data in the mass sideband above 8 GeV. The momentum of the J/Ψ J/Ψ pair in all three directions was tuned for both X and nonresonant production using pythia settings in the second stage to match the data observed in both the sideband and X signal regions.

**Fig. 3:**
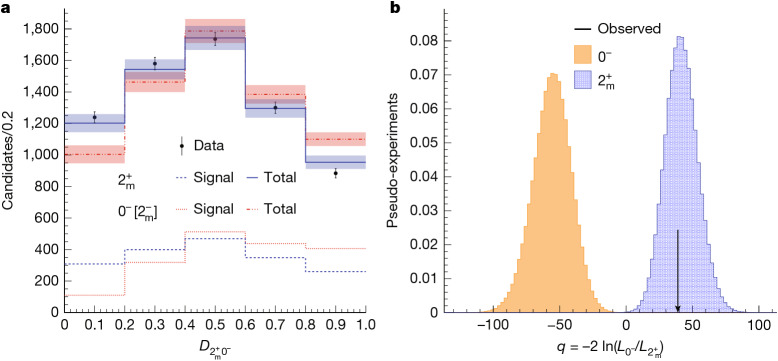
Analysis of angular distributions. **a**, Distributions of ${{\mathcal{D}}}_{{2}_{{\rm{m}}}^{+}{0}^{-}}$ for the 0^−^, ${2}_{{\rm{m}}}^{-}$, and ${2}_{{\rm{m}}}^{+}$ models in the range 6.2 < *m*_4μ_ < 8.0 GeV. Distributions for signal only (dashed) and for signal plus background (solid and dash-dot-dotted) models are compared with the experimental data points with error bars, with uncertainty bands representing post-fit model uncertainties, which are partially correlated with the data. The 0^−^ and ${2}_{{\rm{m}}}^{-}$ distributions are identical apart from systematic uncertainties arising from polarization effects. **b**, Normalized distributions of the test statistic $q=-2{\rm{ln}}({{\mathcal{L}}}_{{0}^{-}}/{{\mathcal{L}}}_{{2}_{{\rm{m}}}^{+}})$ from pseudo-experiments generated under the ${2}_{{\rm{m}}}^{+}$ (blue, right) and 0^−^ (orange, left) hypotheses, with the arrow indicating the observed value *q*_obs_.

We conduct pairwise comparisons between different models characterized by distinct *J*^*PC*^ quantum numbers. Instead of analysing the multidimensional space of angular observables (*θ*_1_, *θ*_2_, *Φ*) directly, we define a single observable, ${{\mathcal{D}}}_{ij}$, constructed from the likelihood ratio between two models labelled *i* and *j*. It relies on quantum-mechanical calculations, as detailed in the section ‘Matrix element approach’ and ref. ^35^. This discriminant ${{\mathcal{D}}}_{ij}$ depends on the angular observables and is optimal for discriminating between the two hypotheses *i* and *j*. The final statistical analysis of the data is carried out using two-dimensional distributions of the events, ${{\mathcal{P}}}_{ij}({m}_{4{\rm{\mu }}},{{\mathcal{D}}}_{ij})$. An example of the predicted distributions for the ${{\mathcal{D}}}_{{2}_{{\rm{m}}}^{+}{0}^{-}}$ observable is presented in Fig. 3a, in which the 0^−^, ${2}_{{\rm{m}}}^{-}$, and ${2}_{{\rm{m}}}^{+}$ models are shown, along with the background and experimental data. Projecting the two-dimensional distribution $({m}_{4{\rm{\mu }}},{{\mathcal{D}}}_{ij})$ onto the single observable ${{\mathcal{D}}}_{ij}$ results in a partial loss of statistical power.

## Quantum number determination

To distinguish alternative models, the test statistic $q=-2{\rm{ln}}({{\mathcal{L}}}_{{J}_{j}^{P}}/{{\mathcal{L}}}_{{J}_{i}^{P}})$ is defined using the likelihood ratio of signal plus background likelihoods for the two signal hypotheses ${J}_{i}^{P}$ and ${J}_{j}^{P}$, based on the two observables $({m}_{4{\rm{\mu }}},{{\mathcal{D}}}_{ij})$, as outlined in the section ‘Statistical analysis’. In a maximum likelihood fit, the likelihood function ${\mathcal{L}}$ is maximized with respect to the nuisance parameters, which include the yields of signal and background processes, as well as constrained parameters that account for systematic uncertainties. These uncertainties encompass variations in mass shapes and in the discriminants used to model both signal and background components, as detailed in the section ‘Systematic uncertainties’.

An example of the observed value of *q*, along with the expected distributions for the ${2}_{{\rm{m}}}^{+}$ and 0^−^ models, is shown in Fig. 3b. The expectations for each model are derived from a large ensemble of pseudo-experiments, with systematic uncertainties from the parameterization included in the fit procedure. These pseudo-experiments are generated according to the observed data shown in Fig. 1, combined with the kinematic distributions specific to each model. Given the observed value of *q*, the ${2}_{{\rm{m}}}^{+}$ model is preferred over 0^−^. All the steps outlined above for testing the spin-parity properties of the X states closely follow the methods and tools developed for the discovery and characterization of the Higgs boson in its four-lepton decay in 2012 (refs. ^35–37^).

**Fig. 4:**
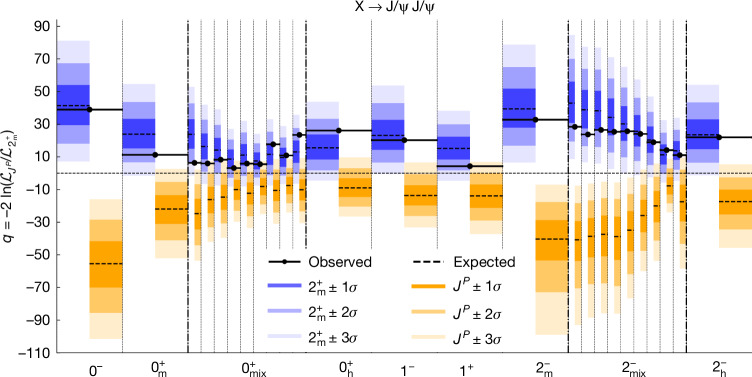
Summary of statistical tests. Distributions of the test statistic *q* for various ${J}_{i}^{P}$ hypotheses tested against the ${2}_{{\rm{m}}}^{+}$ model. The observed *q*_obs_ values are indicated by the black dots. The expected median and the 68.3%, 95.4% and 99.7% confidence level regions for the ${2}_{{\rm{m}}}^{+}$ model (blue, left) and for each of the alternative ${J}_{i}^{P}$ hypotheses (orange, right) are shown. The first entry corresponding to 0^−^ reflects the information shown in Fig. 3b. For 0^+^ and 2^−^ models, 11 points correspond to varying fractions in the mixture of the two structures of interaction.

Following this methodology, all pairs of the eight ${J}_{i}^{P}$ models listed in Table 1 are tested, and in each case involving the ${2}_{{\rm{m}}}^{+}$ model, the latter is favoured. The corresponding results are shown in Fig. 4 and Table 2, in which the probability, *P*-value, and the associated *Z*-score, expressed as the number of standard deviations derived from the one-sided Gaussian tail integral, are shown for an alternative model ${J}_{i}^{P}$ tested against the ${2}_{{\rm{m}}}^{+}$ model.

**Table 2:** Summary of statistical tests

${{\boldsymbol{J}}}_{{\boldsymbol{i}}}^{{\boldsymbol{P}}}$	*P*-value	*Z*-score reject ${{\boldsymbol{J}}}_{{\boldsymbol{i}}}^{{\boldsymbol{P}}}$
0^−^	2.7 × 10^−13^	7.2
${0}_{{\rm{m}}}^{+}$	4.3 × 10^−5^	3.9
${0}_{{\rm{mix}}}^{+}$	1.4 × 10^−2^	2.2
${0}_{{\rm{h}}}^{+}$	3.1 × 10^−9^	5.8
1^−^	8.0 × 10^−8^	5.2
1^+^	4.7 × 10^−3^	2.6
${2}_{{\rm{m}}}^{-}$	4.1 × 10^−12^	6.8
${2}_{{\rm{mix}}}^{-}$	6.5 × 10^−4^	3.2
${2}_{{\rm{h}}}^{-}$	2.2 × 10^−8^	5.5

The *P*-value and the associated *Z*-score are shown for alternative models ${J}_{i}^{P}$, tested against the ${2}_{{\rm{m}}}^{+}$ model. A higher *Z*-score implies that the model is less compatible with the observation.

Building on this observation, a mixture of the ${0}_{{\rm{m}}}^{+}$ and ${0}_{{\rm{h}}}^{+}$ structures of interactions, as well as that of ${2}_{{\rm{m}}}^{-}$ and ${2}_{{\rm{h}}}^{-}$, is tested in 10 equal fractional increments against the ${2}_{{\rm{m}}}^{+}$ model. The ${2}_{{\rm{m}}}^{+}$ model is again preferred. In each case, one of the mixed models shown in Fig. 4, denoted as ${0}_{{\rm{mix}}}^{+}$ or ${2}_{{\rm{mix}}}^{-}$, represents the mixed scenario with the least separation from ${2}_{{\rm{m}}}^{+}$ and is listed in Table 2. A mixture of ${2}_{{\rm{m}}}^{-}$ and ${2}_{{\rm{h}}}^{-}$ contributions does not produce interference because of their differing spin projections along the decay axis, as reflected in the amplitude composition presented in Table 1. We examine both constructive and destructive interference between the ${0}_{{\rm{m}}}^{+}$ and ${0}_{{\rm{h}}}^{+}$ models, by considering both positive and negative relative signs between their contributing amplitudes. Constructive interference results in the smallest deviation from the ${2}_{{\rm{m}}}^{+}$ model, except in the first ${0}_{{\rm{mix}}}^{+}$ step shown in Fig. 4, in which the sign-induced model differences are minimal and destructive interference yields a slightly smaller separation.

Based on the results presented in Fig. 4 and Table 2, the tests for the *J*^*PC*^ = 0^−+^ and 1^−+^ scenarios reject these hypotheses with a significance level exceeding 5 standard deviations when compared with a ${2}_{{\rm{m}}}^{+}$ model. The 2^−+^ scenario, along with higher spin values that have the same *P* and *C* quantum numbers, is excluded at a significance level of 3 standard deviations. This establishes the quantum numbers *P* = +1 and *C* = +1, as shown by the decay final-state particles and their distributions.

The 1^++^ scenario is excluded at more than a 99% confidence level when compared with the ${2}_{{\rm{m}}}^{+}$ model. The *J*^*PC*^ = 0^++^ scenario, when considering a combination of possible amplitudes, is excluded at more than a 95% confidence level when compared with the ${2}_{{\rm{m}}}^{+}$ model. It is important to emphasize that the selected ${2}_{{\rm{m}}}^{+}$ model represents just one possible realization of the *J*^*PC*^ = 2^++^ scenario, and an admixture of other amplitudes could lead to angular distributions resembling those of the 0^++^ or 1^++^ scenarios. The *J* ≥ 3 quantum numbers are still possible, but *J* = 2 is more likely, due to the additional energy needed to achieve a higher angular excitation of the hadronic states with *L* ≥ 2. This makes the *J*^*PC*^ = 2^++^ interpretation preferred for the fully charmed tetraquark states X(6600), X(6900) and X(7100).

## Discussion

The study of tetraquark states has attracted considerable interest because of its potential to provide insight into the structure of the hadronic matter that makes up the world around us^4–8,8–29^. In this study, we have presented the first measurements of the quantum numbers for a recently discovered family of three all-charm tetraquarks, based on data collected by the CMS experiment at the LHC. Our results, summarized in Table 2, favour a *J*^*PC*^ = 2^++^ assignment.

For a system of two constituents, as represented by either model in Fig. 2, an orbital angular momentum of *L* = 1 is excluded by the requirement *P* = +1, whereas higher orbital excitations with *L* ≥ 2 are energetically disfavoured. This makes the *S*-wave (*L* = 0) configuration the most likely. In a molecular scenario, the quark–antiquark pairs are not required to be spin-1 mesons, making a *J* = 2 configuration less likely. This picture agrees with the existing data for all other well-established tetraquark candidates with measured spin, such as the X(3872) and Z_c_(3900)^+^, all of which have *J* < 2 (ref. ^3^). By contrast, a tightly bound ${\rm{c}}{\rm{c}}\bar{{\rm{c}}}\bar{{\rm{c}}}$ tetraquark with a diquark–antidiquark configuration requires both diquarks to be in spin-1 states, which restricts the total spin to *J* = 0 or *J* = 2, making *J* = 2 a natural choice. This spin-1 diquark requirement, however, does not apply to tetraquark candidates with mixed-flavour quark content, a consideration relevant to all previously observed candidates, which contained both heavy and light quarks^3^. Other theoretical considerations also favour *J* = 2 for the tightly bound tetraquark states^18,28^.

This advancement in understanding exotic hadrons was enabled by the study of all-heavy tetraquarks, and it brings us closer to uncovering their internal structure. Although our findings do not definitively distinguish between tightly bound tetraquark and meson–meson molecular models, they provide constraints on the possible internal structures and favour the tightly bound scenario.

## Methods

### Modelling of hadron properties

To investigate the spin-parity properties of the resonant structure within the J/Ψ J/Ψ invariant mass spectrum in the range of 6.2–8.0 GeV, we use an approach designed to minimize model dependence. This approach relies on the observed J/Ψ J/Ψ invariant mass spectrum and the momentum of the J/Ψ J/Ψ system in both transverse and longitudinal directions with respect to the beam, while remaining independent of the polarization of the system by relying solely on decay angular information. For a spin-zero state, polarization is not relevant. For states with nonzero spin, we assume the state is produced unpolarized, but vary the polarization to evaluate potential small residual effects on the decay angular distributions due to detector acceptance.

The analysis uses a model that ensures consistency with the observations made by CMS, by using simulation adjustments to accurately capture the observed transverse and longitudinal motion, and parameters of the resonances and backgrounds extracted from ref. ^32^ and shown in Fig. 1. The background arises from nonresonant contributions, single-parton scattering (SPS) and double-parton scattering, plus an empirical term parameterizing the background near the threshold^32^. The background is parameterized using MC simulation, with adjustments applied to better match the observed data in both the signal and sideband regions.

We start by considering the spin-0 hypothesis for the X states, which are produced without polarization. The helicity amplitudes ${A}_{{\lambda }_{1}{\lambda }_{2}}$ of the two J/Ψ mesons are listed in Table 1. For the pseudoscalar state with *J*^*P*^ = 0^−^, the amplitudes satisfy *A*_++_ = −*A*_−−_ and *A*_00_ = 0. By contrast, for the scalar state with *J*^*P*^ = 0^+^, both *A*_++_ = *A*_−−_ and *A*_00_ amplitudes contribute, with no specific prediction for the relative magnitude of *A*_00_. We adopt the general amplitude approach^35,46,51^, in which the spin-0 state amplitude can be written as a sum of three Lorentz-invariant structures,

1 $$\begin{array}{l}A({{\rm{X}}}_{J=0}\to {V}_{1}{V}_{2})={a}_{1}({q}^{2}){m}_{V}^{2}{{\epsilon }}_{1}^{* }{{\epsilon }}_{2}^{* }+{a}_{2}({q}^{2}){f}_{{\rm{\mu }}\nu }^{* (1)}{f}^{* (2),{\rm{\mu }}\nu }\\ \,+{a}_{3}({q}^{2}){f}_{{\rm{\mu }}\nu }^{* (1)}{\mathop{f}\limits^{ \sim }}^{* (2),{\rm{\mu }}\nu },\end{array}$$

and where the field strength tensor of a vector boson *V*_*i*_ with momentum *q*_*i*_ and polarization vector *ϵ*_*i*_ is defined as ${f}^{(i),{\rm{\mu }}\nu }={{\epsilon }}_{i}^{{\rm{\mu }}}{q}_{i}^{\nu }-{{\epsilon }}_{i}^{\nu }{q}_{i}^{{\rm{\mu }}}$, the conjugate field strength tensor is ${\mathop{f}\limits^{ \sim }}^{(i),{\rm{\mu }}\nu }=1/2{{\epsilon }}^{{\rm{\mu }}\nu {\rm{\alpha }}{\rm{\beta }}}{f}_{{\rm{\alpha }}{\rm{\beta }}}$, and *q* = *q*_1_ + *q*_2_.

As the X decay proceeds by the strong interaction, which conserves parity, the first two terms in equation (1) can be interpreted as interactions involving scalar particles, corresponding to models ${0}_{{\rm{m}}}^{+}$ and ${0}_{{\rm{h}}}^{+}$ with couplings *a*_1_ and *a*_2_, respectively. The third term represents the interaction of a pseudoscalar particle, 0^−^, associated with the coupling *a*_3_. At *m*_4μ_ = 6.9 GeV, ${| {A}_{00}| }^{2}=52 \%$ in model ${0}_{{\rm{m}}}^{+}$ and ${| {A}_{00}| }^{2}=19 \%$ in model ${0}_{{\rm{h}}}^{+}$. In the following, we analyse the two models, ${0}_{{\rm{m}}}^{+}$ and ${0}_{{\rm{h}}}^{+}$, separately. Moreover, we consider their combination, denoted as ${0}_{{\rm{mix}}}^{+}$, in which the relative magnitudes and signs of the couplings *a*_1_ and *a*_2_ are varied. A positive relative sign results in constructive interference, whereas a negative relative sign leads to destructive interference.

**Extended Data Fig. 1:**
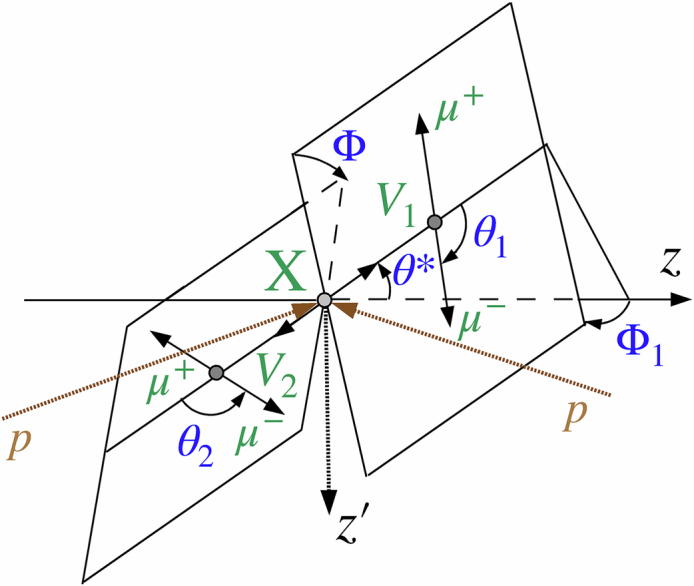
Angular observables. The production and decay of a resonance X in proton collisions pp → X → V_1_V_2_ → 4μ define the angular observables in the centre-of-mass frames of the corresponding particles^35,46^, where the V_1_ and V_2_ refer to the J/Ψ mesons. The *z* axis approximates the proton beam line, while the $z{\prime}$ axis corresponds to the direction of the four-muon system.

The polarization of the spin-1 states varies depending on the production mechanism, in which we consider either unpolarized production or polarization with *J*_*z*_ or ${J}_{{z}^{{\prime} }}=\pm 1$ (ref. ^46^) because quark-initiated production dominates. The *z* and $z{\prime}$ axes are defined in Extended Data Figure 1, where the motion of the four-muon system within the laboratory frame leads to appearance of noncollinear proton collisions and defines the $z{\prime}$ axis, while the *z-*axis approximates the proton beam line. In the decay process, four helicity amplitudes contribute, and their relationships are shown in Table 1. This is equivalent to two Lorentz-invariant structures in the decay amplitude

2 $$\begin{array}{l}A({{\rm{X}}}_{J=1}\to {V}_{1}{V}_{2})={b}_{1}({q}^{2})[({{\epsilon }}_{1}^{* }q)({{\epsilon }}_{2}^{* }{{\epsilon }}_{{\rm{X}}})+({{\epsilon }}_{2}^{* }q)({{\epsilon }}_{1}^{* }{{\epsilon }}_{{\rm{X}}})]\\ \,+{b}_{2}({q}^{2}){{\epsilon }}_{\alpha \mu \nu \beta }{{\epsilon }}_{{\rm{X}}}^{\alpha }{{\epsilon }}_{1}^{* ,\mu }{{\epsilon }}_{2}^{* ,\nu }{\widetilde{q}}^{\beta },\end{array}$$

where $\widetilde{q}={q}_{1}-{q}_{2}$ and *ϵ*_X_ is the polarization vector of the spin-1 resonance X. The *b*_1_ and *b*_2_ are the couplings to a vector state with *J*^*P*^ = 1^−^ and an axial vector with *J*^*P*^ = 1^+^, respectively.

The polarization of the spin-2 states also depends on the production mechanism, in which we consider either unpolarized production or polarization with *J*_*z*_ or ${J}_{{z}^{{\prime} }}=0$ or ±2 (ref. ^46^) because gluon-initiated production is expected to dominate. In the decay process, all nine helicity amplitudes ${A}_{{\lambda }_{1}{\lambda }_{2}}$ contribute. This corresponds to the Lorentz-invariant structures in the decay amplitude found in refs. ^35,46^. There are two degrees of freedom in the 2^−+^ case, and we use the ${2}_{{\rm{m}}}^{-}$ and ${2}_{{\rm{h}}}^{-}$ models, corresponding to ${g}_{8}^{(2)}\ne 0$ and ${g}_{10}^{(2)}\ne 0$ in refs. ^35,46^, respectively. The ${2}_{{\rm{m}}}^{-}$ model corresponds to *A*_++_ = −*A*_−−_, with decay angular distributions identical to those of the 0^−^ case. For ${2}_{{\rm{h}}}^{-}$, we have *A*_+0_  = *A*_0+_ = −*A*_−0_ = −*A*_0−_, with decay angular distributions identical to those of the 1^−^ case.

We use a single representative model for the *J*^*PC*^ = 2^++^ state, corresponding to model ${g}_{1}^{(2)}={g}_{5}^{(2)}\ne 0$ denoted as ${2}_{{\rm{m}}}^{+}$ in refs. ^35,46^. This ${2}_{{\rm{m}}}^{+}$ model is chosen because the composition of ${A}_{{\lambda }_{1}{\lambda }_{2}}$ amplitudes represents all possible polarizations, in particular those that are unique for spin-2, *A*_+−_ and *A*_−+_ and the tensor structure of interactions is minimal, avoiding the inclusion of higher-dimension operators. At *m*_4μ_ = 6.9 GeV, $2{| {A}_{++}| }^{2}=9 \%$, ${| {A}_{00}| }^{2}=21 \%$, $4{| {A}_{+0}| }^{2}=47 \%$ and $2{| {A}_{+-}| }^{2}=23 \%$ in model ${2}_{{\rm{m}}}^{+}$. Therefore, if the data are consistent with ${J}^{P}={2}_{{\rm{m}}}^{+}$ and not with the other models, it will provide an unambiguous determination of *J* ≥ 2. A higher spin scenario (*J* > 2) could also be possible, and it would exhibit angular distributions similar to those of *J* = 2 with the same parity^35,46^.

As the *J*^*PC*^ = 2^++^ state has four degrees of freedom in the amplitude composition, three other possibilities could be chosen that lead to the same observable decay distributions as the ${0}_{{\rm{m}}}^{+}$, ${0}_{{\rm{h}}}^{+}$ and 1^+^ states. Interference between the corresponding 2^++^ amplitude tensor structures is also possible. Therefore, from a purely decay-based analysis, if the data are consistent with either the 0^++^ or 1^++^ model, we cannot rule out a general 2^++^ model. Although analysing polarization information through production-sensitive angular distributions could help resolve this ambiguity, this analysis is more challenging due to uncertainty in the production mechanism. If the resonance is produced unpolarized, such an analysis would not provide additional information. In this study, we check production-sensitive angular distributions for consistency with an unpolarized case, whereas a more detailed analysis is left for future work.

Determining the form factors associated with the tensor structure of interactions, represented by *a*_*i*_(*q*^2^) in equation (1) for spin-0, *b*_*i*_(*q*^2^) in equation (2) for spin-1, or equivalent ${g}_{i}^{(2)}({q}^{2})$ in refs. ^35,46^ for spin-2, with ${q}^{2}={m}_{4{\rm{\mu }}}^{2}$, is nontrivial and relies on the model of strong interactions. Nevertheless, it is completely separable from the computation of the angular distributions, given a particular tensor structure of interactions. Furthermore, interference between the resonances could result in a complex alteration of the angular distributions. Nonetheless, assuming that all three resonances possess identical quantum numbers and coupling constants, the angular distributions remain unaffected by interference effects observed in the mass spectrum. Thus, in this study, we use an empirical approach to analyse the four-muon mass spectrum observed in the data, separating it from the analysis of angular distributions. This makes our analysis independent of the form factor model. The form factors and interference effects are integrated into the observed mass spectrum, as shown in Fig. 1.

### Angular observables

The angular information used in the analysis is shown in Extended Data Fig. 1 (refs. ^35,46^). Two production axes are defined, corresponding to the two production mechanisms, either the direct short-distance production in parton collisions or the fragmentation of a single parton into the hadron. The z-axis is parallel to the beam line within the frame boosted along the beam line from the laboratory frame, in which the longitudinal momentum of X is zero, potentially reflecting polarization in the two-parton collisions. The *z*′-axis aligns with the direction of the X momentum, reflecting potential polarization in the single-parton fragmentation scenario.

The decay angles *Φ*, *θ*_1_ and *θ*_2_ are defined with reference to Fig. 2. The production angles θ* and *Φ*_1_, or alternatively ${\theta }^{{\prime} * }$ and *Φ*_1_′, are defined with respect to the *z-* or *z*′-axis, respectively. The angle θ* is defined between the *z*-axis and the X decay axis in its rest frame, whereas *Φ*_1_ is the angle between the first J/Ψ decay plane and the production plane, defined by the *z*-axis and the X decay axis. Extended Data Fig. 1 shows only θ* and *Φ*_1_, whereas ${\theta }^{{\prime} * }$ and *Φ*_1_′ are defined analogously. These production angles are not used directly in the analysis to avoid dependence on the production model, but they are checked for consistency.

**Extended Data Fig. 2:**
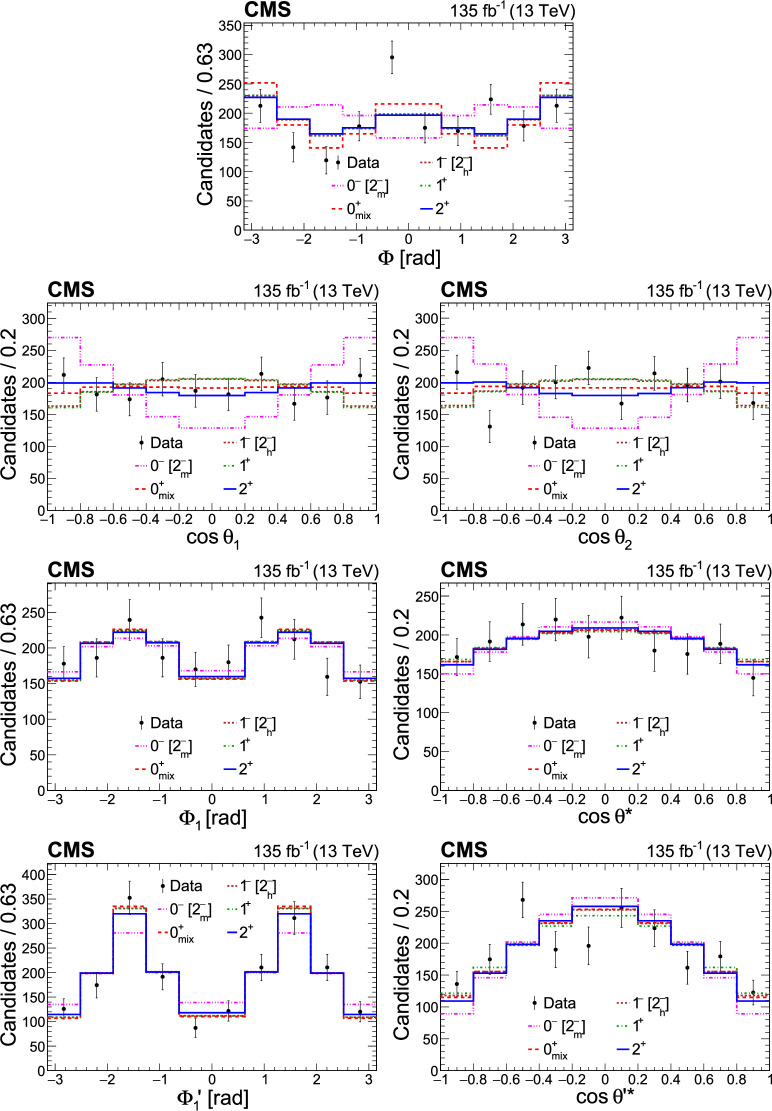
Angular distributions. Distribution of the decay angles: Φ (upper row), $\cos {\theta }_{1},\cos {\theta }_{2}$ (second row), production angles defined with respect to axis *z*: ${\Phi }_{1},\cos {\theta }^{* }$ (third row), and defined with respect to axis $z{\prime}$: ${\Phi }_{1}^{{\prime} },\cos {\theta }^{{\prime} * }$ (lower row) in the range 6.2 < *m*_4μ_ < 8.0 GeV presented together with the five ${J}_{i}^{P}$ signal models. The background is subtracted from the data, based on the expected distributions, and systematic uncertainties are not incorporated in these plots. The 0^−^ and ${2}_{m}^{-}$ distributions are identical, as are those of 1^−^ and ${2}_{h}^{-}$.

The distributions of the decay and production angles in the range 6.2 < *m*_4μ_ < 8.0 GeV are presented in Extended Data Fig. 2, together with the five signal models shown. It is important to note that the one-dimensional angular distributions in Extended Data Fig. 2 do not capture all the information accessible to the optimal discriminant. Correlations between angles are lost in the projections; for instance, the models *J*^*PC*^ = 1^−+^ and 1^++^ cannot be distinguished in each projection, but they can be separated using the optimal discriminants, which preserve all angular correlations. Furthermore, Extended Data Fig. 2 shows all events in the range 6.2 < *m*_4μ_ < 8.0 GeV together, without accounting for the correlation between the angular distributions and *m*_4μ_, or the variation in signal purity with *m*_4μ_ due to the resonance structure appearing above the background. As a result, the separation power shown in these illustration plots is considerably diminished compared with that in the full analysis.

To eliminate the dependence on the initial polarization of nonzero-spin states, the production angles are excluded from the data analysis. The assumption is made that the resonances are produced unpolarized, although the variation of this polarization along the production axes is allowed for states with nonzero spin. This allows for the examination of any residual effects stemming from polarization dependence due to detector acceptance effects. Extended Data Fig. 2 shows that the production angular distributions are consistent with unpolarized resonance states along both the *z-* and *z*′-axes.

### Matrix element approach

The analysis of the multidimensional distributions ${\mathcal{P}}({\theta }_{1},{\theta }_{2},\varPhi ,{m}_{4{\rm{\mu }}})$ is complicated by the complex description and the nontrivial effects of detector reconstruction. Rather than using the three angular observables directly, we construct a single observable that effectively projects the angular information onto one dimension and is optimal for distinguishing between two hypotheses, ${J}_{i}^{P}$ and ${J}_{j}^{P}$. Using a matrix element likelihood approach^35,46,51^, a kinematic discriminant is formulated based on the ratio of probabilities for hypotheses ${J}_{i}^{P}$ and ${J}_{j}^{P}$:

3 $${{\mathcal{D}}}_{ij}({m}_{4{\rm{\mu }}},{\boldsymbol{\Omega }})=\frac{{{\mathcal{P}}}_{j}({m}_{4{\rm{\mu }}},{\boldsymbol{\Omega }})}{{{\mathcal{P}}}_{i}({m}_{4{\rm{\mu }}},{\boldsymbol{\Omega }})+{{\mathcal{P}}}_{j}({m}_{4{\rm{\mu }}},{\boldsymbol{\Omega }})},$$

where ${{\mathcal{P}}}_{i}$ is the normalized probability based on the matrix element squared for a given hypothesis ${J}_{i}^{P}$. These matrix elements are computed within the same quantum-mechanical formalism as used for the generation of MC events, as detailed in section ‘Angular analysis’.

**Extended Data Fig. 3:**
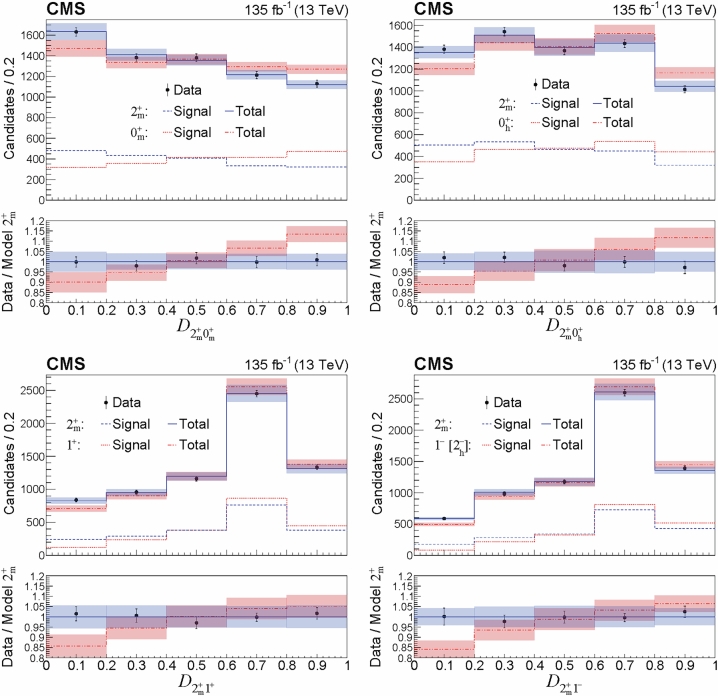
Optimal observables. Distributions of ${{\mathcal{D}}}_{ij}$ that are optimal for separating the ${2}_{m}^{+}$ model against the ${0}_{m}^{+}$ (upper left), ${0}_{h}^{+}$ (upper right), 1^−^ (lower left), and 1^+^ (lower right) models in the range 6.2 < *m*_4μ_ < 8.0 GeV. Distributions for signal only (dashed) and for signal plus background (solid and dash-dot-dotted) models are compared to the experimental data points with error bars, with uncertainty bands representing post-fit model uncertainties, which are partially correlated with the data. The 1^−^ and ${2}_{h}^{-}$ distributions are identical, apart from systematic uncertainties arising from polarization effects. The lower panels display the ratios of the data and of the model predictions to the mean expectations from the ${2}_{m}^{+}$ model.

In equation (3), we use only the decay angles ${\boldsymbol{\Omega }}=\{\cos {\theta }_{1},\cos {\theta }_{2},\varPhi \}$. The analysis is conducted using the two-dimensional distributions of $({m}_{4{\rm{\mu }}},{{\mathcal{D}}}_{ij})$. Production information, including $\{\cos {\theta }^{* },{\varPhi }_{1}\}$ or $\{\cos {\theta }^{{\prime} * },{\varPhi }_{1}^{{\prime} }\}$, can be incorporated into a future analysis if a study of the resonance polarization is desired. The distributions of the discriminants, which were calculated to assess alternative models in comparison to the ${2}_{{\rm{m}}}^{+}$ model, are presented in Fig. 3 and Extended Data Fig. 3 for all models defined without considering amplitude mixtures. The kinematic distributions in Fig. 3 and Extended Data Figs. 2 and 3 have also been separately examined in three different ranges of *m*_4μ_, each corresponding to one of the three resonance structures shown in Fig. 1. The data remain consistent with the ${2}_{{\rm{m}}}^{+}$ model across the full mass range as well as within each of the three individual intervals.

The final statistical analysis of the data is carried out using two-dimensional distributions of events, ${{\mathcal{P}}}_{ij}({m}_{4{\rm{\mu }}},{{\mathcal{D}}}_{ij})$, where the predicted *m*_4μ_ distribution is modelled in the same way as in Fig. 1 following ref. ^32^, and the ${{\mathcal{D}}}_{ij}$ distribution is obtained in bins of 0.05 GeV in *m*_4μ_ from the detailed MC simulation discussed earlier in section ‘Angular analysis’. For any given slice of *m*_4μ_, only five bins of ${{\mathcal{D}}}_{ij}$ are used, meaning that the MC simulation can accurately predict probability distributions using an affordable number of simulated events.

### Systematic uncertainties

In the maximum likelihood fit introduced in section ‘Quantum number determination’ and further detailed in the next section, the likelihood function is maximized with respect to the nuisance parameters, which include those representing systematic uncertainties. The fit results encompass systematic variations in the parameterization of both signal and background models. The yields of the signal and each of the background processes are treated as free parameters, to be fully determined by the fit to the data. Further variations are categorized into two groups: variations in mass shapes and variations in discriminants, applied to both signal and background components.

The study of systematic variations in mass shapes was detailed in ref. ^32^, which includes resonance parameterization, resolution and efficiency for signal components, as well as different models for background components. All these variations in mass shapes were incorporated into the two-dimensional analysis presented here, through the variation of ${{\mathcal{P}}}_{k}({m}_{4{\rm{\mu }}})$ in equation (4). Therefore, all uncertainties associated with the parameterization of the *m*_4μ_ spectrum as detailed in ref. ^32^ are included in the analysis.

One of the uncertainties arises from the unknown angular distributions of the background contribution near the kinematic threshold^32^. To allow maximum flexibility, the discriminant parameterization of this contribution, ${T}_{ijk}({{\mathcal{D}}}_{ij}| {m}_{4{\rm{\mu }}})$ in equation (4), is expressed as the sum of the SPS background model and the two signal models under investigation, with the relative contributions of all three components determined by freely floating nuisance parameters. This approach is intended to accommodate a broad range of hypotheses for the threshold contribution, including possible variations of the nonresonant background and potential resonance excitations.

Other systematic variations in discriminants account for possible inconsistencies in *p*_T_ and *p*_*z*_ distributions between data and MC simulation. The *p*_T_ and *p*_*z*_ distributions are validated and tuned through comparisons between data and simulation in both the sideband region (8.0–9.0 GeV) and the signal region (6.2–8.0 GeV). Residual discrepancies in the discriminants due to mismodelling of *p*_T_ and *p*_*z*_ are addressed through nuisance parameters, which allow for alternative discriminant parameterizations of ${T}_{ijk}({{\mathcal{D}}}_{ij}| {m}_{4{\rm{\mu }}})$ in equation (4). Similarly, uncertainties in the kinematic distributions of the nonresonant background components are evaluated through comparisons between data and MC simulations in the sideband region, in which a satisfactory agreement is observed. Remaining discrepancies in the discriminants are handled through nuisance parameters that permit alternative discriminant parameterizations of ${T}_{ijk}({{\mathcal{D}}}_{ij}| {m}_{4{\rm{\mu }}})$.

The nominal discriminant parameterization of all spin-1 and spin-2 resonances assumes no polarization. However, to evaluate the impact of detector acceptance effects on the results, we introduce an alternative parameterization that assumes polarization of spin-1 and spin-2 resonances along either the *z*- or *z*′-axis. This approach models polarized production arising from either two-parton collisions or single-parton fragmentation.

### Statistical analysis

We perform a binned extended maximum likelihood fit in which the probability density function is a sum of contributions from all signal and background processes implemented in the Combine tool (v.10.0.2) (ref. ^52^). This method mirrors the approach used to determine the spin-parity quantum numbers of the Higgs boson at the LHC^53^, as detailed in ref. ^35^. Each process *k* is characterized by a probability density function ${{\mathcal{P}}}_{ijk}$, used to analyse signal hypotheses *i* and *j*. This function depends on two observables, *m*_4μ_ and ${{\mathcal{D}}}_{ij}$, and is defined as a template binned in a 36 × 5 grid:

4 $${{\mathcal{P}}}_{ijk}({m}_{4{\rm{\mu }}},{{\mathcal{D}}}_{ij})={{\mathcal{P}}}_{k}({m}_{4{\rm{\mu }}}){T}_{ijk}({{\mathcal{D}}}_{ij}| {m}_{4{\rm{\mu }}}),$$

where ${{\mathcal{P}}}_{k}({m}_{4{\rm{\mu }}})$ represents the probability density function of the invariant mass *m*_4μ_, which is independent of the hypotheses being tested. The probability density *T*_*ijk*_ is a normalized function of ${{\mathcal{D}}}_{ij}$ given a specific value of *m*_4μ_, obtained from simulation and including systematic variations through alternative distributions, as described above. We assume the same quantum numbers and couplings for all signal resonances, enabling the use of a shared *T*_*ijk*_ for parameterizing the signal.

To distinguish between alternative models, the test statistic $q=-2{\rm{ln}}({{\mathcal{L}}}_{{J}_{j}^{P}}/{{\mathcal{L}}}_{{J}_{i}^{P}})$ is defined using the ratio of signal plus background likelihoods for the two signal hypotheses. The likelihood is maximized with respect to the nuisance parameters, which include the yields of signal and background (bkg) processes and constrained parameters describing the systematic uncertainties. To quantify the consistency of the observed test statistic *q*_obs_ with the model ${J}_{i}^{P}$, the probability $P=P(q\le {q}_{{\rm{obs}}}\,| \,{J}_{i}^{P}+\,\mathrm{bkg})$ is determined under the signal-plus-background hypothesis using pseudo-experiments. This probability is then translated into a *Z*-score, representing the number of standard deviations using the one-sided Gaussian tail integral.

The consistency of the observed data with the alternative signal hypothesis (${J}_{j}^{P}$) is assessed from $P(q\ge {q}_{{\rm{obs}}}\,| \,{J}_{j}^{P}+\,{\rm{bkg}})$. The sign is positive if the tail extends away or negative if it extends towards the median of the other hypothesis. The CL_s_ criterion^54,55^, defined as ${{\rm{CL}}}_{{\rm{s}}}=P(q\ge {q}_{{\rm{obs}}}\,| \,{J}_{j}^{P}+\,\mathrm{bkg}\,)$/$P(q\ge {q}_{{\rm{obs}}}\,| \,{J}_{i}^{P}+\,{\rm{bkg}}) < \alpha ,$ is used for the final inference of whether a particular alternative hypothesis ${J}_{j}^{P}$ is excluded or not with respect to a reference hypothesis ${J}_{i}^{P}$ at a given confidence level (1 − *α*). Figure 3b shows example *q* distributions for the ${2}_{{\rm{m}}}^{+}$ and 0^−^ models, obtained from repeated pseudo-experiments simulating the expected experimental outcome.

The pairs of spin-parity models are tested among the 0^−^, ${0}_{{\rm{m}}}^{+}$, ${0}_{{\rm{h}}}^{+}$, 1^−^, 1^+^, ${2}_{{\rm{m}}}^{-}$, ${2}_{{\rm{h}}}^{-}$, and ${2}_{{\rm{m}}}^{+}$ hypotheses. In all tests involving the ${2}_{{\rm{m}}}^{+}$ model, it is preferred. Therefore, these tests are presented in Fig. 4 and Extended Data Table 1, which is an extended version of Table 2. The pairwise tests between the other models do not provide any additional useful information, as the data frequently show inconsistencies with both models. In the case of *J*^*PC*^ = 0^++^ and 2^−+^ scenarios, additional tests are conducted to account for a possible mixture of two tensor structures, as outlined in section ‘Quantum number determination’.

It is important to note that the ${2}_{{\rm{m}}}^{+}$ model represents only one specific realization of the *J*^*PC*^ = 2^++^ hypothesis. In practice, a mixture of amplitudes corresponding to the 2^++^ state, as listed in Table 1, could produce angular distributions that resemble those expected for 0^++^ or 1^++^, apart from ${2}_{{\rm{m}}}^{+}$. As a result, even if the true particle is a 2^++^ state, the *P*-values reported in Extended Data Table 1 may not remain fully consistent with the ${2}_{{\rm{m}}}^{+}$ model under such admixtures. These mixed scenarios can be explored in future analyses. Taking this into account, all observed data distributions are found to be compatible with the *J*^*PC*^ = 2^++^ hypothesis and show deviations from the predictions of alternative *J*^*PC*^ assignments, with confidence levels summarized in Extended Data Table 1.

## Online content

Any methods, additional references, Nature Portfolio reporting summaries, source data, extended data, supplementary information, acknowledgements, peer review information; details of author contributions and competing interests; and statements of data and code availability are available at 10.1038/s41586-025-09711-7.

## Figures and Tables

**Extended Data Table 1:**
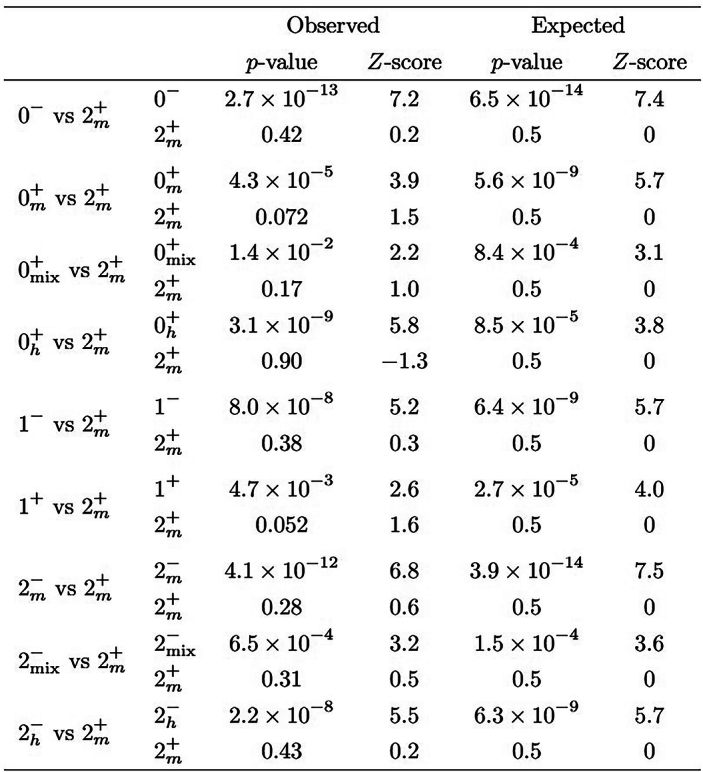
Results from hypothesis test for pairs of spin-parity models

This is an extended version of Table 2. The expected P-value is presented based on the assumption of the scenario of ${2}_{m}^{+}$. Results with *Z* > 5 have been derived through Gaussian extrapolation.

## Data Availability

Release and preservation of data used by the CMS Collaboration as the basis for publications is guided by the CMS data preservation, re-use and open access policy (https://opendata.cern.ch/record/415).

## Code availability

The CMS core software is publicly available at GitHub (https://github.com/cms-sw/cmssw).
